# Multiple origins of downy mildews and mito-nuclear discordance within the paraphyletic genus *Phytophthora*

**DOI:** 10.1371/journal.pone.0192502

**Published:** 2018-03-12

**Authors:** Tyler B. Bourret, Robin A. Choudhury, Heather K. Mehl, Cheryl L. Blomquist, Neil McRoberts, David M. Rizzo

**Affiliations:** 1 Department of Plant Pathology, University of California, Davis, Davis, California, United States of America; 2 Plant Pathology Department, University of Florida, Gainesville, Florida, United States of America; 3 California Department of Food and Agriculture, Sacramento, California, United States of America; Agriculture and Agri-Food Canada, CANADA

## Abstract

Phylogenetic relationships between thirteen species of downy mildew and 103 species of *Phytophthora* (plant-pathogenic oomycetes) were investigated with two nuclear and four mitochondrial loci, using several likelihood-based approaches. Three *Phytophthora* taxa and all downy mildew taxa were excluded from the previously recognized subgeneric clades of *Phytophthora*, though all were strongly supported within the paraphyletic genus. Downy mildews appear to be polyphyletic, with graminicolous downy mildews (GDM), brassicolous downy mildews (BDM) and downy mildews with colored conidia (DMCC) forming a clade with the previously unplaced *Phytophthora* taxon totara; downy mildews with pyriform haustoria (DMPH) were placed in their own clade with affinities to the obligate biotrophic *P*. *cyperi*. Results suggest the recognition of four additional clades within *Phytophthora*, but few relationships between clades could be resolved. Trees containing all twenty extant downy mildew genera were produced by adding partial coverage of seventeen additional downy mildew taxa; these trees supported the monophyly of the BDMs, DMCCs and DMPHs but suggested that the GDMs are paraphyletic in respect to the BDMs or polyphyletic. Incongruence between nuclear-only and mitochondrial-only trees suggests introgression may have occurred between several clades, particularly those containing biotrophs, questioning whether obligate biotrophic parasitism and other traits with polyphyletic distributions arose independently or were horizontally transferred. Phylogenetic approaches may be limited in their ability to resolve some of the complex relationships between the “subgeneric” clades of *Phytophthora*, which include twenty downy mildew genera and hundreds of species.

## Introduction

Oomycetes (Phylum Oomycota, Kingdom Straminipila) are eukaryotic microbes that include many important pathogens of plants [1]. In particular, species in the genus *Phytophthora* are among the greatest socioeconomic threats to agriculture and natural ecosystems [2,3]. Also important is the group of oomycetes known as the downy mildews, which comprise twenty genera and a large portion of described oomycete species [4,5]. *Phytophthora* and the downy mildews, with the early-diverging *Halophytophthora*, *Nothophytophthora* and *Phytopythium* constitute a monophyletic clade known as the Peronosporaceae s.l. [1,4,6]. Nearly every member of the Peronosporaceae is associated with plants, either as a parasite (necrotrophic, biotrophic or hemibiotrophic) or as a saprotroph of dead plant tissue.

All downy mildews are considered obligate biotrophic plant parasites, meaning they only grow in association with living host tissue and cannot be cultured on artificial media [7]. A few *Phytophthora* species are not culturable, only known from plant hosts and therefore also considered to be obligate biotrophs [2,8,9]. Obligate biotrophic plant parasitism arose multiple times within the oomycetes, being found in one group besides the Peronosporaceae s.l.: the white rusts of the Albuginales [1]. How many times the trait arose within the Peronosporaceae is unknown; downy mildews are not necessarily thought to constitute a single monophyletic clade. Phylogenetic studies have shown inconsistent results [10,11], but downy mildews are typically placed into either one or two monophyletic clades nested within the genus *Phytophthora* (rarely from a downy mildew-*Phytophthora* common ancestor) (Table 1). The obligate biotrophic *Phytophthora* species have been considered distinct from downy mildews based on morphological characteristics; to date none of these unculturable species, including *Phytophthora cyperi*, *P*. *leersiae*, *P*. *lepironiae* and *P*. *polygoni* have been subjected to DNA sequencing. As a result, the phylogenetic distribution of obligate biotrophic plant parasitism within the oomycetes remains unresolved.

**Table 1:** Relevant phylogenetic studies including both *Phytophthora* and downy mildews.

Author(s)	Year	# of loci	Nuclear, Mito-chond-rial, or Com-bined data?	# of *Phyto*. taxa	*Phyto*. clades represented	# of DM taxa	# of BDM genera	#of DMCC genera	# of DMPH genera	# of GDM genera	*Phyto*. Mono-phyletic?	DMs mono-phyletic?	*Phyto*. clade(s) DMs either share a most recent common ancestor with or are within
Cooke et al. [12]	2000	1	N	47	1–8,12	1		1			N		4
Cooke et al. [13]	2002	1	N	13	1,2,4,6–8	11		1			N	Y	1,4
Riethmüller et al. [14]	2002	1	N	9	1,2,4,6,7	63	1	2	5	1	Y	N	1,2,4,6,7
Voglmayr [15]	2003	1	N	11	1,2,4,6–9	10	2	2			N	Y	4
Göker et al. [16]	2007	4	C	18	1–10, 12	50	2	2	7	3	N	Y	1
Thines et al. [17]	2008	1	M	18	1–10, 12	67	2	2	7	6	N	N	5; 9
Thines et al. [11]	2008	2	C	18	1–10, 12	56	2	2	7	5	N	N	1; 1,4,5,12
Uzuhashi et al. [18]	2010	2	M	17	1–3, 5–10, 12	47	1	2	5	1	N	Y	1
Lara and Belbahri [19]	2011	1	N	19	1,2,4,6,7,9	4	1	1	1		N	N	1
Runge et al. [20]	2011	7	N	117	1–10, 12	2	1	1			N	Y	4
Sharma et al. [21]	2015	393	N	4	1,2,7,8	2	1		1		N	N	1; 1,2,7,8
Ye et al. [22]	2016	293	N	7	1,2,4,7,8,10	3	1	1	1		N	Y	1,2,4
McCarthy and Fitzpatrick [23]	2017	2280	N	18	1–3,5–8,10,15	3	1		1		N	N	15; 1
This study (118 taxa; Fig 1, S1 Fig)	2018	6	C	103	1–10, 12–15	13	2	2	2	1	N	N	15; 14
This study (135 taxa; Fig 2)	2018	6	C	103	1–10, 12–15	30	2	2	8	8	N	N	15; 14

**Fig 1:**
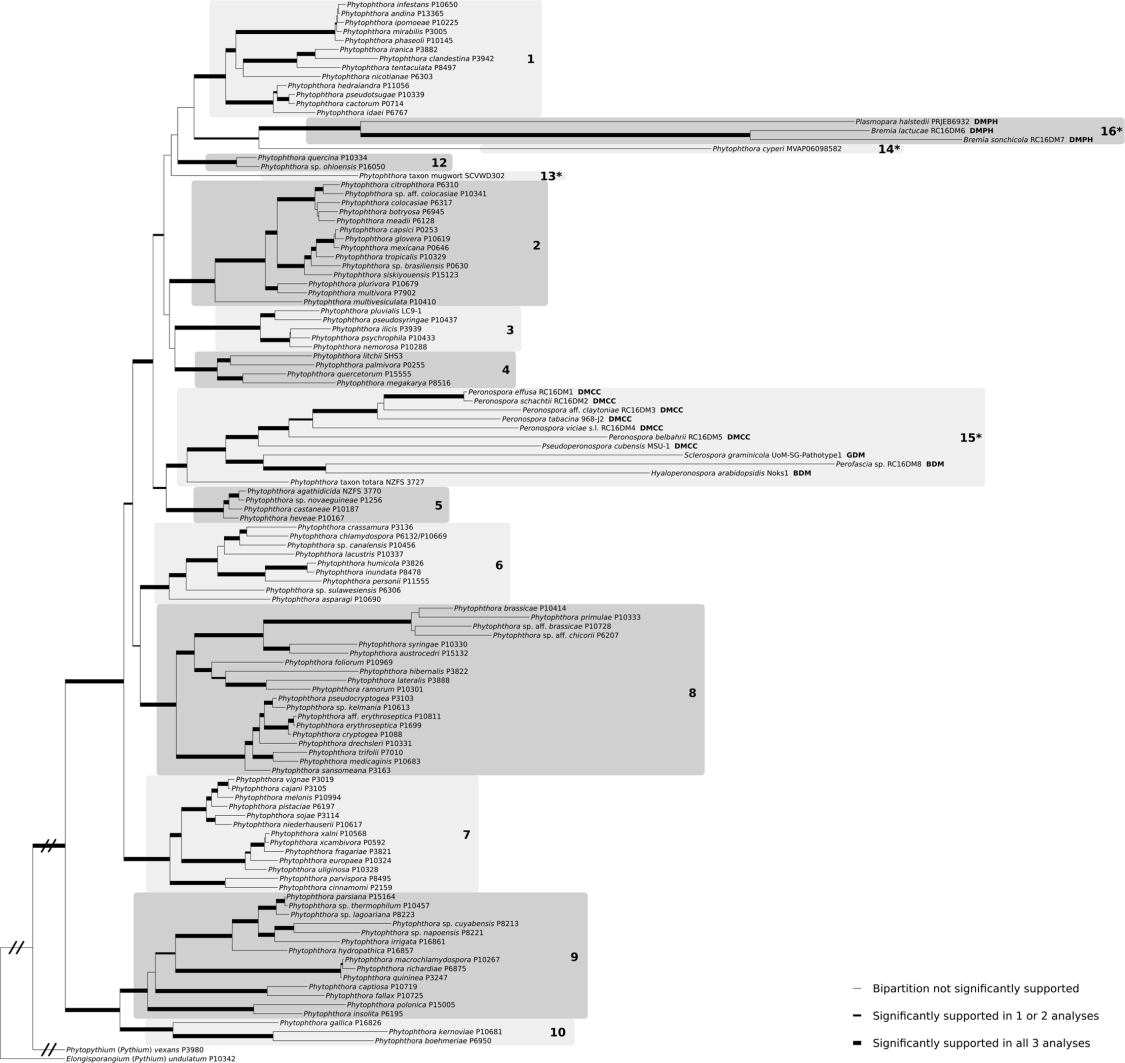
Most likely tree from a 118-taxon alignment of *Phytophthora* and downy mildew species. Tree generated with 200 heuristic best tree searches using Garli 2.01. Bipartitions receiving significant support in Garli, MrBayes or PhyloBayes analyses are thickened and bipartitions receiving significant support in all three analyses are greatly thickened. Significant support was defined as bootstrap value ≥ 0.70 or posterior probability ≥ 0.95. Slashes indicate branches shortened for display purposes. The subgeneric clades of *Phytophthora* [12,24,25] are numbered in bold; additional clades appearing in the tree are numbered 13–16 and denoted with an asterisk. Clade 11, containing *P*. *lilii* [24] could not be included in the current study. DM, downy mildew; BDM, brassicolous DM; DMCC, DM with colored conidia; DMPH, DM with pyriform haustoria; GDM, graminicolous DM. *Phytophthora* strain numbers beginning with “P” refer to the World Oomycete Genetic Resource Collection. Figure created with TreeGraph2 and Inkscape.

Clades are illustrated in Fig 1. *Phyto*., *Phytophthora*. DM, downy mildew.

**Fig 2:**
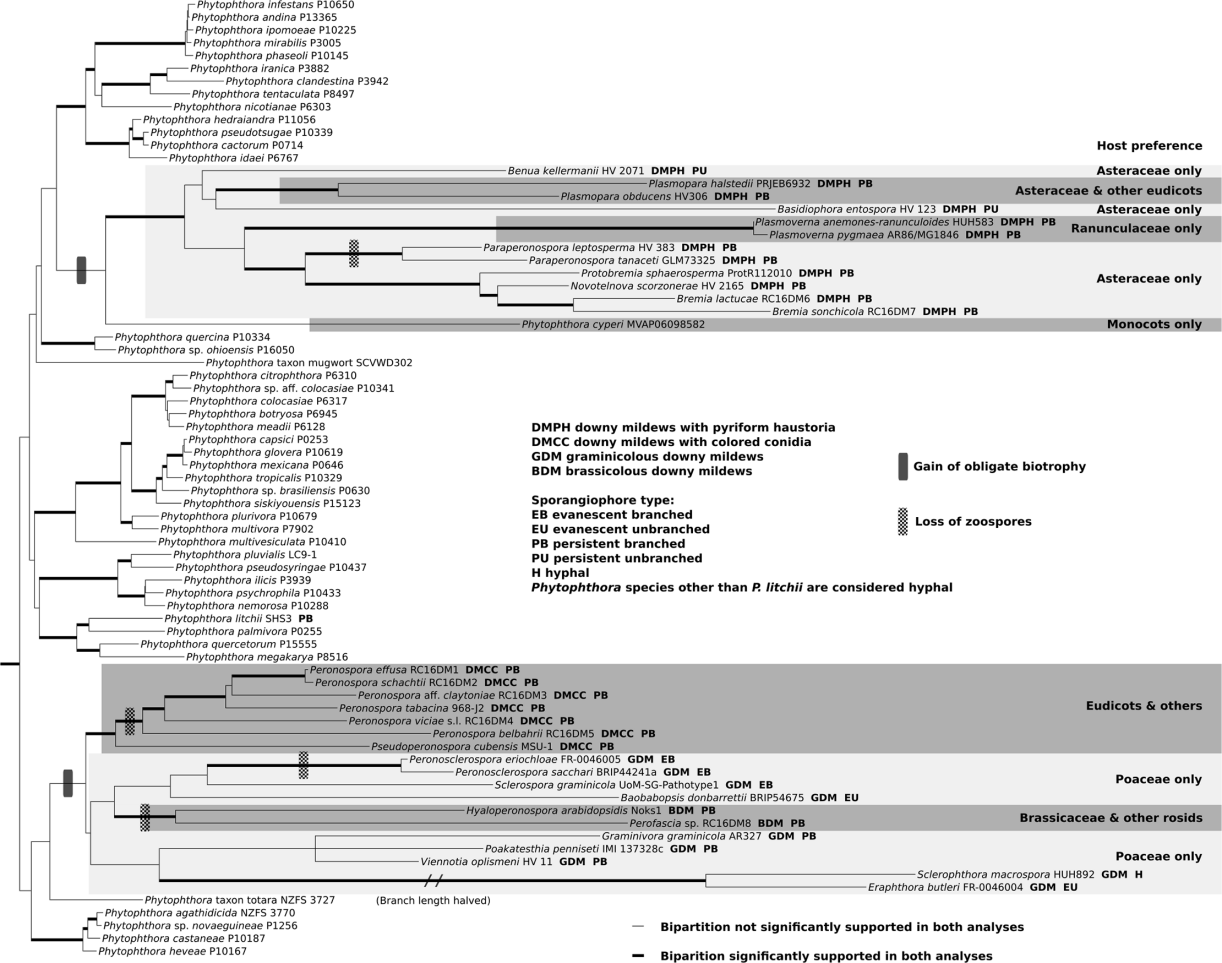
Most likely tree from 135-taxon alignment of *Phytophthora* and downy mildew species, excerpt. Maximum likelihood (ML) tree, omitting outgroups and *Phytophthora* clades 6–10 from the figure. Thickened branches correspond to significantly supported bipartitions in both ML (Garli) and Bayesian likelihood (MrBayes); significant support was defined as bootstrap value ≥ 0.70 or posterior probability ≥ 0.95. Sporangiophore characteristics and host preferences are indicated for downy mildew taxa and *P*. *cyperi*. The length of the branch connecting the common ancestor of *Eraphthora* and *Sclerophthora* was halved for display purposes. DM, downy mildew; BDM, brassicolous DM; DMCC, DM with colored conidia; DMPH, DM with pyriform haustoria; GDM, graminicolous DM. Figure created with TreeGraph2 and Inkscape.

Early oomycete classifications tended to group downy mildews genera together, but Dick et al. [26] considered the grass-infecting graminicolous downy mildews (GDM) to be sufficiently distinct from the other, mostly eudicot-infecting downy mildews to be placed in a separate order, the Sclerosporales. As a result of recent molecular phylogenetic work, all downy mildew genera are considered members of the Peronosporaceae s.l., and have been placed into four natural groups, which may or may not correspond to phylogenetic clades [5] (Table 1). These groups are downy mildews with pyriform haustoria (DMPH), downy mildews with colored conidia (DMCC), brassicolous downy mildews (BDM) and GDM. While DMPH and DMCC are groups united by synapomorphic morphological characteristics, BDM and GDM are grouped by plant host preference, though some BDM species occur on other eudicots. While grouped based on their distinctive haustoria, most DMPH genera specialize on Asteraceae. The ability to infect the above-ground tissue of grasses is rare for any member of the Peronosporaceae s.l. outside of the GDM, and therefore the phylogenetic position of the *P*. *cyperi*, which has hosts in the Poaceae [27], in relation to the GDM is of particular interest. Above ground, obligate biotrophic parasitism on monocots is limited within the oomycetes only to the GDM, *Peronospora destructor*, *Peronospora fugitai*, *Phytophthora cyperi*, *P*. *leersiae* and *P*. *lepironiae*. The relationships between any of the downy mildews and the genus *Phytophthora* are unresolved, though *Phytophthora* species are the other organisms to which downy mildews appear most closely related (Table 1).

Obligate biotrophy is an exceptional trait with a limited distribution among major groups of fungal-like organisms. The process is thought to begin with an intimate association of a parasite and a host. Hemibiotrophs might either extend the biotrophic phase, reducing or abolishing the necrotrophic phase, or some combination of the two. If the parasite is able to elude host defenses successfully enough to spend most or all of its life in symbiosis (e.g. with vertical transmission), the host can be relied on solely for nutrients, making certain metabolic pathways of the parasite redundant and eventually lost, resulting in obligatory parasitism [28–31]. Similar losses of metabolic pathways appear to have taken place within the Kingdom Fungi in pathogens of other organisms and in plant symbionts [32,33], suggesting obligate biotrophy is a consequence of a sustained and intimate symbiosis and present in multiple eukaryotic kingdoms. Very fastidious organisms and obligate biotrophs may represent a continuum; species of *Peronospora*, *Sclerospora* and *Sclerophthora* have rarely been reported as axenically culturable under specific conditions [34–36].

The first phylogenetic inference for the genus *Phytophthora* used nuclear ITS rDNA and identified ten numbered clades [12], with an eleventh corresponding to *P*. *quercina* evident in retrospect (Table 1) and later recognized by [24]. As new species were added to the genus, a multi-locus data set was needed to accurately place some early-diverging species, first using nuclear [25] and then combined nuclear and mitochondrial loci [37], necessitating an update of the definition of the clades beyond “ITS clades.” Currently, the subgeneric clades can be described as the largest monophyletic clades retrieved from multi-locus phylogenies that receive significant statistical support and within which most relationships also receive strong statistical support. Blair et al. [25] was unsure about what to do with single-species that did not fit into other clades (orphan taxa), placing *P*. *quercina* in clade 4, but questioning whether the species might constitute its own clade. More recently two orphan taxa, *P*. *stricta* and *P*. *lilii* have each been assigned novel clades based on their exclusion from other clades in multi-locus phylogenies [24,38]. The combination of exceptional diversity and obligate biotrophy has resulted in slower progress towards a similar set of sequence data for downy mildews, with efforts instead focused on broader coverage of barcoding loci and establishing monophyletic genera [4,39–46]. Without multi-locus sequence coverage from more than a few downy mildew taxa available in GenBank, the *Phytophthora* and downy mildew phylogenetic data sets are largely incompatible. Additionally, the accelerated evolution of downy mildews presents methodological difficulties. As a result, genus-wide phylogenies of *Phytophthora* often omit downy mildews, despite evidence that downy mildews represent a group of highly-derived *Phytophthora* species.

The potential for reticulate evolution within oomycete lineages may underlie some of the methodological difficulties and incongruence that characterize phylogenetic inference of relationships within the group. Hybridization appears to have occurred frequently within the oomycetes, especially *Phytophthora* [47], creating abundant opportunities for the horizontal transfer of genes, albeit within a more phylogenetically limited context than the horizontal gene transfer documented by [48]. Because reticulate evolution cannot be correctly modeled by traditional phylogenetic methods, artifacts may be generated when those methods are applied to reticulating clades.

**Fig 3:**
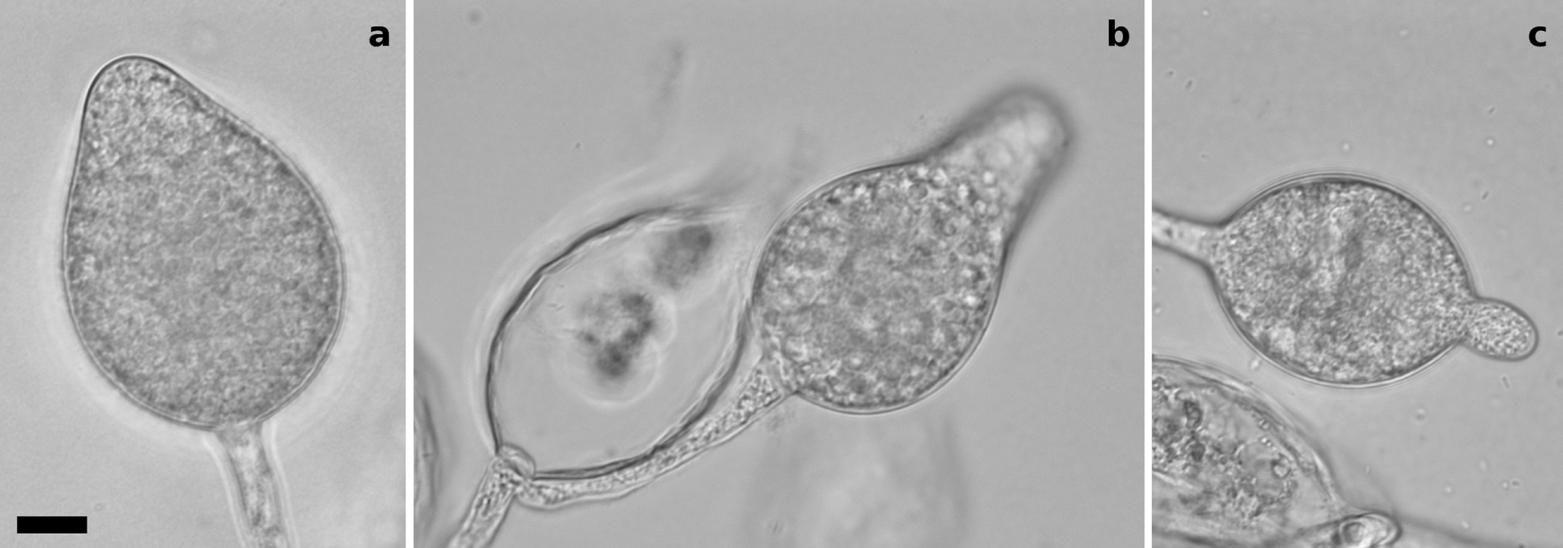
Asexual sporulation in *Phytophthora* taxon mugwort strain SCVWD302. Sporangia are non-papillate, non-caducous and variable in shape, borne in tight sympodial clusters on long stalks. The distal tips of the sporangia may swell with cytoplasm, producing ovoid to obpyriform shapes or sometimes forming a beak-like protuberance. A, ovoid sporangium; B, external proliferation with evacuated sporangium and obpyriform sporangium; C, ellipsoid sporangium with protuberance. DIC micrographs with Leica DM5000B at 1000X. Scale bar = 10 μm.

The goal of this study was to understand the evolutionary relationships between the genus *Phytophthora* and the downy mildews. Modern systematic efforts emphasize the importance of conserving only monophyletic taxa. With a data set with sequences from all known downy mildew genera and a phylogenetically wide *Phytophthora*, we wanted to understand if sporangial structure in the downy mildews was related to phylogeny and whether the four downy mildew groups constituted clades. With the sequence information from an obligate biotrophic *Phytophthora* available for the first time we wanted to know if obligate biotrophy arose within *Phytophthora* s.l. independently from the downy mildews. We used the combined nuclear and mitochondrial data set recommended by [37] to place downy mildew taxa in relation to *Phytophthora* taxa. To augment a selection of six loci from this *Phytophthora* data set (Table 2), twelve additional species of *Phytophthora* were added from the GenBank nucleotide collection and four species from GenBank genome-sequencing data (S1 Table), including *P*. taxon totara, a phylogenetically distinct species from New Zealand [49] and the downy mildew-like *P*. *litchii* [22]. New to this study were sequences from a sample of *P*. *cyperi*, a species first described in 1903 but never before phylogenetically placed and *P*. taxon mugwort (Fig 3), a novel species recently isolated by authors of this manuscript. Added to these 103 *Phytophthora* species were thirteen downy mildew species corresponding to eight samples collected in northern and central California and five BioProjects for which genome-sequencing data was publicly available. At least one member of all four downy mildew groups was able to be included in this 118-taxon, full-coverage data set (Table 3, S1 Table).

**Table 2:** Summary of loci used for phylogenetic inference.

	Locus	Genome	Coding	bp
**LSU**	D1-D7 regions of large subunit (28S) ribosomal DNA	N		1299
**btub**	beta-tubulin	N	Y	1137
**cox2**	cytochrome c oxidase subunit 2	M	Y	678
**nad9**	NADH dehydrogenase subunit 9	M	Y	552
**rps10**	ribosomal protein S10	M	Y	321
**secY**	sec-independent transporter protein (ymf16)	M	Y	729

N = nuclear, M = mitochondrial. bp refers to the number of positions in alignments used for inference.

**Table 3:** Downy mildew taxa included in the current study.

Downy mildew taxon	Group	Source
*Baobabopsis donbarrettii*	GDM	N
*Basidiophora entospora*	DMPH	N
*Benua kellermanii*	DMPH	N
*Bremia lactucae*	DMPH	S
*Bremia sonchicola*	DMPH	S
*Eraphthora butleri*	GDM	N
*Graminivora graminicola*	GDM	N
*Hyaloperonospora arabidopsidis*	BDM	G
*Novotelnova scorzonerae*	DMPH	N
*Paraperonospora leptosperma*	DMPH	N
*Paraperonospora tanaceti*	DMPH	N
*Perofascia* sp.	BDM	S
*Peronosclerospora eriochloae*	GDM	N
*Peronosclerospora sacchari*	GDM	N
*Peronospora belbahrii*	DMCC	S
*Peronospora* aff. *claytoniae*	DMCC	S
*Peronospora effusa*	DMCC	S
*Peronospora schactii*	DMCC	S
*Peronospora tabacina*	DMCC	G
*Peronospora viciae* s.l.	DMCC	S
*Plasmopara halstedii*	DMPH	G
*Plasmopara obducens*	DMPH	N
*Plasmoverna anemones-ranunculoides*	DMPH	N
*Plasmoverna pygmaea*	DMPH	N
*Poakatesthia penniseti*	GDM	N
*Protobremia sphaerosperma*	DMPH	N
*Pseudoperonospora cubensis*	DMCC	G
*Sclerophthora macrospora*	GDM	N
*Sclerospora graminicola*	GDM	G
*Viennotia oplismeni*	GDM	N

G, GenBank genome-sequencing data; N, partial coverage obtained from the GenBank nucleotide collection; S, this study from Californian samples. The thirteen “G” and “S” taxa (shaded) were included in all phylogenetic inference, while the remaining “N” taxa are only featured in the 135-taxon data set used for Fig 2 and S3 Fig. DM, downy mildew; BDM, brassicolous DM; DMCC, DM with colored conidia; DMPH, DM with pyriform haustoria; GDM, graminicolous DM.

A second data set used a “supermatrix” approach to sample all twenty downy mildew genera by adding partial coverage of an additional seventeen taxa. While phylogenetic data sets with large amounts of missing data typically result in lower support values and have even been demonstrated to result in biased results under certain circumstances [50], it has also been shown that trees inferred from most real-world multi-locus data sets are unlikely to suffer greatly from missing data [51], and that adding phylogenetically distinct taxa, even at low coverage, can actually improve inference [52]. This study is the first time a molecular phylogeny has featured every extant genus of downy mildew.

## Results

The alignment files and tree output files for all analyses can be found at TreeBASE.org under accession S21585. The results of the phylogenetic inference from the 118-taxon data set are illustrated in Fig 1. Most bipartitions were significantly supported in all analyses, including strong support for each of the subgeneric clades of *Phytophthora*. Deeper branching patterns between the clades were not well-supported and often varied between analyses. Exceptions included strong support for *Phytophthora*+downy mildews, *Phytophthora* clades 1–8,12–14+downy mildews, and *Phytophthora* clades 1–5,12–14+downy mildews. Although the eleven included previously recognized subgeneric clades of *Phytophthora* were recovered with strong support, the results suggest at least four additional clades should be recognized to account for the downy mildews and three *Phytophthora* taxa that could not be placed into the other clades. These clades were provisionally given numbers 13–16 (Fig 1). The downy mildews were found not to be monophyletic; DMPHs formed a separate clade, while DMCCs, *Sclerospora* (representing GDMs) and BDMs were placed in a strongly supported clade with *P*. taxon totara. *Phytophthora* clade 1, DMPHs (clade 16), *P*. *cyperi* (clade 14), *P*. *quercina*/*ohioensis* (clade 12) and *P*. taxon mugwort (clade 13) often clustered, but relationships were not consistent or consistently well-supported; there was a similar association between *Phytophthora* clade 5 and *P*. taxon totara+DMCC+GDM+BDM (clade 15). The trees inferred from the larger 135-taxon alignment were largely congruent with the first (Fig 2). The remaining six genera of DMPH formed a monophyletic clade with *Bremia* and *Plasmopara*. No significant support was found for the early diverging branches of the DMPH or the GDM and GDM were paraphyletic with respect to BDM. In the MrBayes analysis (not illustrated, available at TreeBASE S21585), *Baobabopsis* formed a clade with *Phytophthora* clade 12 rather than with the rest of the GDMs and BDMs; this contrasted the topology in the most likely tree inferred by Garli (Fig 2), where *Baobabopsis* clustered with the other GDMs. The same placement of *Baobabopsis* away from the other GDMs was also seen in the MrBayes analysis of the mitochondrial-only tree derived from the 135-taxon data set (S3 Fig).

Investigations into substitution saturation suggested none of the 16 subsets needed to be excluded (S6 Table). A tree inferred that did exclude the five subsets with the highest index of substitution saturation (I_SS_) (S1 Fig) was congruent with the well-supported clades of Fig 1, suggesting the inclusion or exclusion of these most saturated subsets was not a source of bias. Trees with outgroups and/or long-branched taxa removed were inferred to look for evidence of long branch attraction (LBA) using methods commonly called long branch extraction [53] did not show evidence of LBA.

The nuclear-loci-only and mitochondrial-loci-only trees inferred from the smaller data set (S2 Fig) showed three putative cases of deep mito-nuclear discordance. First, the DMPH were placed within *Phytophthora* clade 1 in the nuclear-only tree, while in the combined and mitochondrial-only trees, DMPH and clade 1 were each strongly supported as separate and monophyletic. *Phytophthora cyperi* was placed as a sister to *Sclerospora graminicola* in the nuclear-only tree, while in the combined and mitochondrial-only trees it was not placed into any other clade and was sister to DMPH. Third, one of the outgroup taxa, *Phytopythium vexans* was placed in *Phytophthora* clade 7 in the mitochondrial-only tree, but was well-supported as separate from *Phytophthora* in combined and nuclear-only trees. A fourth case of deep nuclear-only and mitochondrial-only incongruence was observed with the larger data set; *Baobabopsis donbarrettii*, a GDM, was placed in clade 12 in the mitochondrial-only tree (S3 Fig). In the nuclear-only tree with added taxa *Phytophthora cyperi* was related to a polyphyletic GDM as an early diverging member of the clade containing *Baobabopsis*, *Peronosclerospora* and *Sclerospora*. The topology of DMCC did not change between nuclear-only and mitochondrial-only trees, but the DMPH exhibited polytomies in the mitochondrial-only tree. The relationships between BDM and GDM were resolved in both nuclear-only trees but contained polytomies in mitochondrial-only trees, and BDM were not monophyletic in the mitochondrial-only trees.

Many of the subgeneric clades were not monophyletic in single-locus phylogenies, with low support values and early-diverging lineages or subclades forming polytomies (S4–S9 Figs); only from beta-tubulin could a phylogeny be inferred that resembled the combined analysis, although clade 8 was polyphyletic (S5 Fig). The signal placing *P*. *cyperi* close to *Sclerospora* came from the LSU; in the btub-only tree, *P*. *cyperi* was placed in clade 4, and not within any other clades in the four mitochondrial, single-locus trees (S6–S9 Figs). Clade 4 was not monophyletic in the LSU-only tree either, with *P*. taxon mugwort placed in the clade with low support (S4 Fig). The placement of the DMPH within clade 1 was only retrieved in the btub-only single-locus tree. Although the cox2-only tree placed *Phytopythium vexans* within *Phytophthora* when rooted with *Elongisporangium*, it was the *Phytopythium* secY locus that strongly resembled that of *P*. *cinnamomi* (S9 Fig), underlying the topology seen in the mitochondrial-only trees of S2 and S3 Figs. Like the results from the six loci combined, the remaining four loci supported *Phytopythium* as a secondary outgroup to *Phytophthora*+downy mildews when rooted with *Elongisporangium*. Also in the secY-only tree, *P*. *hydropathica*, normally a member of clade 9, was well-supported as a member of clade 7. *Peronospora* and *Pseudoperonospora* were not contained within *Phytophthora* when the cox2-only tree was rooted with *Elongisporangium* (S6 Fig).

## Discussion

Although no evolutionary scenarios can be ruled out, our analysis suggests multiple origins of obligate biotrophy within the *Phytophthora* para-genus, with the most parsimonious scenario suggesting two independent origins: once in the lineage leading to the BDMs, DMCCs and GDMs, and a second time in the lineage leading to *P*. *cyperi* and the DMPHs (Fig 2). Only Göker and Stamatakis [10] and McCarthy and Fitzpatrick [23] found the same separation of DMs into two groups, with the DMPHs apart from the other downy mildews, although relatively few prior *Phytophthora*+downy mildew phylogenies included the DMPHs (Table 1). The close and well-supported relationship between the BDMs, DMCCs and GDMs with the southern hemisphere, foliar, conifer-associated *Phytophthora* taxon totara [49] is notable. Although downy mildews arising from a foliar *Phytophthora* ancestor presents a reasonable scenario, none has yet been found on a non-angiosperm host [54]. The topology of the BDM and GDM (Fig 2), with paraphyletic GDM suggests BDM are the result of a host jump from a graminicolous ancestor. While GDM appear confined to Poaceae hosts [4,54], *Hyaloperonospora* has diversified onto non-Brassicaceae hosts [39]. The topology of the DMPH suggests the common ancestor had specialized on the Asteraceae, with host jumps to the Ranunculaceae by ancestors of *Plasmoverna* and jumps to other eudicots by some members of *Plasmopara*.

Phylogenies that contain both long and short branches present methodological difficulty; taxa on long branches may be artificially attracted to or repelled from each other [55]. The scheme used for the Garli and MrBayes phylogenetic analyses, in which codon positions were partitioned separately is likely to produce a relatively robust tree in spite of potential long-branch effects by allowing separate model parameters for the differently-evolving codon positions [56]; this aggressive partitioning was supported as the best-fitting scheme with PartitionFinder. The novel method of modeling molecular evolution employed by PhyloBayes, in which site-specific heterogeneity is accounted for (the CAT model) may also yield favorable results for trees with long branches [57]. While covarion models [58] that attempt to incorporate heterotachy are available in both PhyloBayes and MrBayes, sufficient run convergence could not be reached when these options were invoked. The data used did not appear to suffer from substitution saturation so greatly as to interfere with inference of proper topology (S6 Table) and a tree inferred with the five most saturated subsets omitted did not significantly differ from trees inferred from the entire alignment (S1 Fig). Removing taxa from the alignment or adding long-branched leaves back to the tree one-by-one also did not greatly affect results, suggesting the phylogenetic placement of long-branched taxa was not negatively influenced by other long branches.

The secY locus from one of the outgroup taxa, *Phytopythium vexans* strain P3980, was nearly identical (741/744 bp) to that of the *Phytophthora cinnamomi* isolate included in the study, P2159 (S9 Fig, accession JF770612.2). The P3980 secY sequence completely matches several *P*. *cinnamomi* secY accessions (JF770595.2, JF770592.2, JF770583.1) strongly suggesting, especially owing to similarity in third codon position, that the P3980 secY sequence is not the result of convergent evolution, but that P3980, as presented by Martin et al. [37], is chimeric, either due to biological or other reasons. The P3980 sequences do not have GenBank accessions associated with them, only available from TreeBASE, and there are no other *Phytopythium* secY accessions for comparison. The methods of long branch extraction were used to investigate possible long branch attraction, and accordingly, all analyses of the multi-locus data were re-run without the two outgroup taxa with no significant topological differences were found without the outgroups. This indicates that the putatively chimeric nature of P3980 was not negatively affecting the phylogenetic inference, outside of the placement of *Phytopythium vexans* in S2 and S9 Figs. Removing the *Pythium* s.l. outgroups from the mitochondrial-only trees of S2 and S3 Figs, or running the mitochondrial-only analysis without secY did not otherwise significantly change the topology. Because of this, and also towards the goal of re-analyzing and expanding on the work of Martin et al. [37], we did not seek out alternative outgroup taxa for this study. Nevertheless, due to previously described issues of long-branch attraction, substitution saturation and rare taxon sampling, future studies may wish to add taxa from *Halophytophthora* and *Nothophytophthora*, which appear to be more closely related to *Phytophthora* and the downy mildews than either of the outgroups used in this study [6]. Five out of the six loci used in this study were obtained from an isolate of *Nothophytophthora caduca* using the conditions outlined, but the nad9 locus could not be amplified under any conditions, possibly due to a lack of synteny.

All obligate biotrophic taxa exhibited long branches, with *Bremia sonchicola* appearing to be the most derived from the *Phytophthora*-downy mildew common ancestor in Fig 1, and *Sclerophthora macrospora* in Fig 2; the long branches as displayed on the phylograms are indicative of relatively rapid evolution since the *Phytophthora*-downy mildew common ancestor(s). The long branch exhibited by the biotrophic *P*. *cyperi* suggests the two traits are connected; this is a pattern observed in unrelated groups and confirmed by the relatively short branch of *P*. *litchii*, which, while resembling a downy mildew, is not an obligate biotroph [59]. Only three culturable *Phytophthora* species, *P*. *primulae*, *P*. sp. aff. *brassicae* and *P*. sp. aff. *chicorii* appeared more derived than *Peronospora effusa*, which was the least-derived obligate biotroph in Figs 1 and 2. *P*. *primulae* appears to be relatively host-specific, like most obligate biotrophs, but is a root-parasite, a rare trait among the obligate biotrophic *Peronosporaceae* s.l. [2,54]. This unique combination of traits suggests members of clade 8b (which is also characterized by reticulate evolution [47]), should be targeted for comparative genomic investigation of the evolution of host-specialization and biotrophy, much like *P*. *litchii* [22].

**Fig 4:**
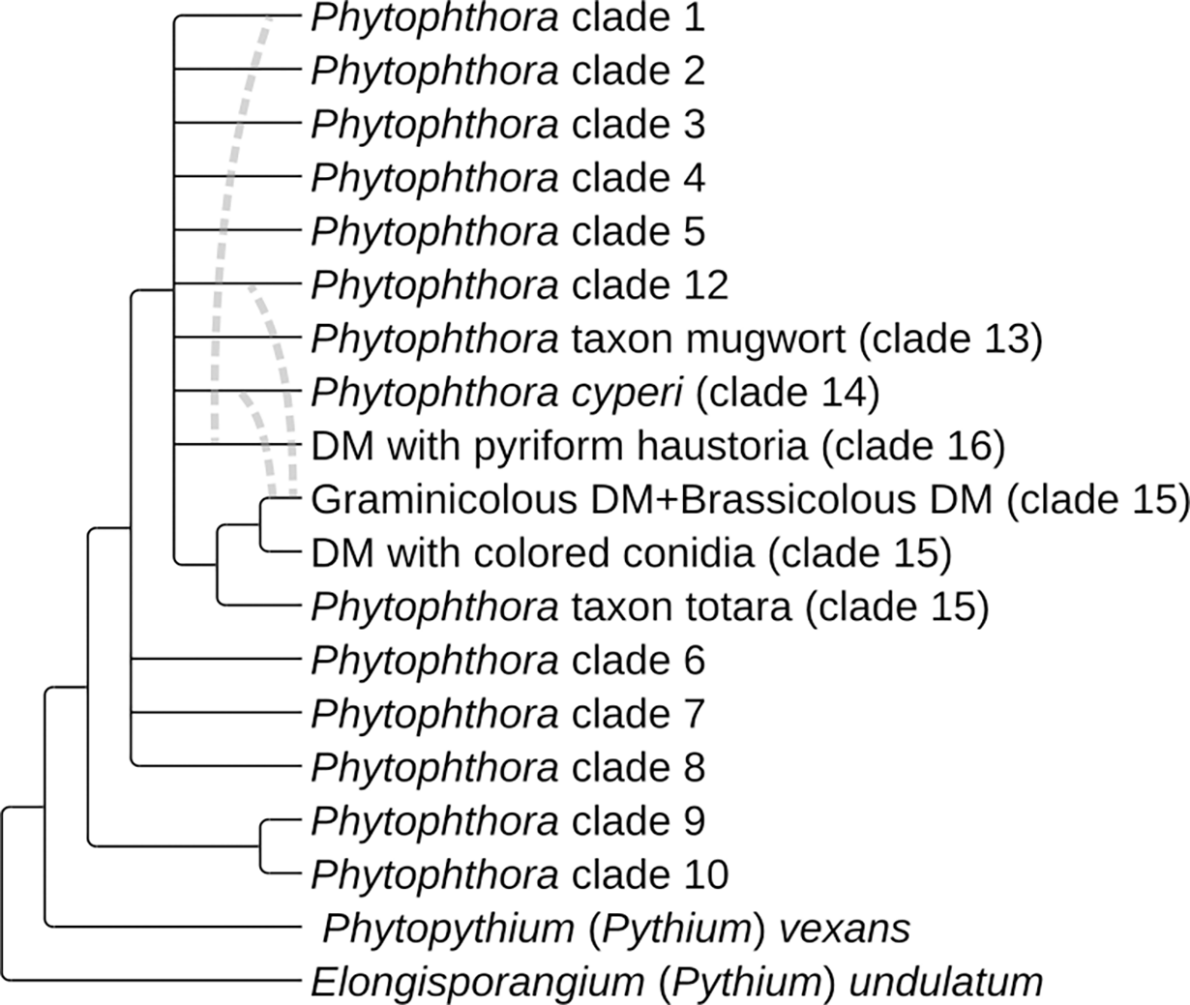
Cladogram with proposed relationships between the genus *Phytophthora*, the four downy mildew groups, and outgroups. Dotted lines indicate possible incongruence between subgeneric clades suggested by nuclear-only and mitochondrial-only trees (S2 and S3 Figs). Clade 11, containing *P*. *lilii* could not be included in the current study, but appears related to clades 6–8 [24]. *Phytophthora stricta* [38] appears to occupy an additional subgeneric clade. Figure created with TreeGraph2 and Inkscape.

The deep incongruence observed between nuclear-only and mitochondrial-only trees (Fig 4, S2 and S3 Figs), if interpreted as biological signals and not the result of phylogenetic artifacts, offers possible alternative explanations for some of the most notable results, including the suggestion that both monocot-specificity and obligate biotrophy are traits that have been converged upon. Instead, the incongruence raises the possibility that these traits have been moved horizontally. Was a particular gene or a series of genes that allowed for more intimate host associations horizontally transferred, followed by parallel losses of saprotrophic abilities? Ye et al. [22] found the genome of the downy mildew-like *Phytophthora litchii* to be more characteristic of *Phytophthora* s.s., but it had a “streamlined” quality similar to downy mildew genomes, with more tightly spaced genes and a reduction of certain gene families. It is notable that despite their apparent independent origins, members of the DMPH and the other downy mildews share many traits not typically associated with *Phytophthora* s.s., such as downy mildew-like sporangiophores and the ability to infect seed tissue. A major question still lingers: did all proto-*Phytophthora* species have the genetic capacity to become obligate biotrophs, and, by chance and circumstance only two or more lineages did, or do the biotrophic lineages share something (either introgressed or converged-on) that the *Phytophthora*-like lineages lack? This same question can be extended farther back in time to include the Albuginales and the other Peronosporomycete lineages.

The mito-nuclear discordance also highlights the limitations of phylogenetic methods that assume divergence only [60], urging systematists to look forward to coalescent approaches that may be able to incorporate such deep discordance between loci into species trees. Although several multi-species coalescent methods were attempted with the 118-taxon data set, the validation of these approaches in light of long branches and heterotachy was beyond the scope of this manuscript. Besides the incongruence highlighted in S2 and S3 Figs, most of the subgeneric clades, while otherwise monophyletic in all trees, exhibited some degree of mito-nuclear discordance, suggesting complex histories for many clades of *Phytophthora*. This within-clade incongruence has been previously reported [37,61,62]. Other possible sources of mito-nuclear discordance besides hybridization include incomplete lineage sorting and phylogenetic artifacts due to insufficient data or violation of model assumptions.

Sporangiophore characteristics appear to be related to phylogeny to some extent; GDM with persistent, branching sporangiophores formed a clade in both maximum likelihood and Bayesian trees, while *Peronosclerospora*, *Sclerospora* and *Baobabopsis*, all of which have evanescent sporangiophores, formed a clade in the maximum likelihood tree (Fig 2). DMPH genera with unbranched sporangiophores appeared to be early-diverging lineages, though these relationships were not well-supported. In contrast, *Eraphthora* and *Sclerophthora*, which do not have morphologically similar sporangiophores, were strongly supported as a clade in all trees. Possible reticulations between members of GDM and *Phytophthora* species implies that all evolutionary possibilities leading to their unique morphologies should be considered, especially considering the morphological similarity of *Phytophthora* and *Sclerophthora*.

While direct germination of sporangia appears to be a facultative trait possessed by most *Phytophthora* species [2], it is the only option for *Hyaloperonospora arabidopsidis*, which has lost functionality of many zoospore-associated genes [63]; a similar pattern was observed in the genome of *Peronospora tabacina* [64]. Complete absence of zoospore release has been considered an advanced feature among downy mildews [7,54], a change that has been seen as an adaptation to an above-ground, drier existence as a downy mildew, and a distinctive enough trait to distinguish two pairs of downy mildew genera: *Pseudoperonospora* from *Peronospora* (DMCC) and *Sclerospora* from *Peronosclerospora* (GDM) [4]. If the genera *Hyaloperonospora*, *Paraperonospora*, *Perofascia*, *Peronosclerospora* and *Peronospora* are considered non-zoospore-producing and zoospore production an ancestral state [7], four independent losses could be mapped onto the tree (Fig 2). Persistent, branched sporangiophores releasing sporangia that only directly germinate, characteristics of *Hyaloperonospora* and *Perofascia* (BDM), *Paraperonospora* (DMPH), and *Peronospora* (DMCC) (which together contain the majority of described downy mildew species) appear to have been converged on at least three times, though more study is needed to rule out other possibilities.

It is systematically untenable to have twenty genera contained within another under the current nomenclatural code [65]. The clearly paraphyletic *Phytophthora* (1876) is predated by the genera *Peronospora* (1837), *Bremia* (1843) and *Basidiophora* (1869), but is arguably the most important of the twenty-one genera in question, with regulations or recommendations often applied to the genus rather than individual species. The close relationship between DMPH (containing *Bremia*, which has priority) and *Phytophthora* clade 1, containing *P*. *infestans*, the type species, in nuclear-only trees suggests a monophyletic *Phytophthora* may contain as few as five species (S2 and S3 Figs). A possible solution would redefine *Phytophthora* as a para-genus, defining *Phytophthora* as a clade that is known to be explicitly paraphyletic in respect to other clades, or not defining it as a clade at all [60]. This would allow the currently recognized genera to remain, all of which appear to be monophyletic except *Phytophthora*. While this overtly paraphyletic system is not currently allowed and controversial, it is the de-facto system, since *Phytophthora* has been known to be potentially paraphyletic since the first molecular phylogeny was published [12,66].

## Conclusions

Relationships within the Peronosporaceae s.l. remain enigmatic. This study highlights the importance of adding sequences from previously unsampled taxa, which may have a greater impact on difficult phylogenies than adding additional loci [67]. The 135-taxon data set produced the first phylogenies to contain all currently recognized downy mildew genera, a significant contribution to the overall understanding of downy mildew evolution. According to our findings, two of the four downy mildew groups are not monophyletic, one arising from within the other. The diversity of taxa included in the data set illuminated several previously unrecognized, strongly supported subgeneric clades within *Phytophthora* s.l.; two clades contained downy mildew taxa and one contained both *Phytophthora* and downy mildews. Incorporating an obligate biotrophic *Phytophthora* species for the first time, the trait appears to have evolved at least twice within the Peronosporaceae, and, owing to the obligate biotrophic Albuginales, at least three times within the Peronosporomycetes. As this manuscript was being prepared, preliminary phylogenetic placement of *Phytophthora polygoni* suggests the existence of an additional, uncharacterized subgeneric clade and an additional acquisition of obligate biotrophy within *Phytophthora* s.l.

When studying the evolution of the Peronosporaceae s.l., all scenarios must be on the table, from convergence towards particular traits, to widespread introgression to horizontal transfer from other kingdoms [48]. This study incorporated a range of phylogenetic tools, particularly those that may alleviate long-branch effects, highlighting areas of phylogenetic consensus in *Phytophthora* s.l. (Fig 4). However, discordant phylogenetic signals from mitochondrial and nuclear genomes preclude the inference of a phylogeny that could be considered definitive. In light of genealogical incongruence exacerbated by long-branch difficulties, the conflicting results of nearly twenty years of combined *Phytophthora* and downy mildew phylogenies (Table 1) can be largely reconciled, but only by accepting that the methods used cannot discern portions of their true history. It is not known whether the appropriate methods or the organismal “missing links” required to fully comprehend these evolutionary scenarios are currently in the hands of any researchers.

## Methods

### Isolation of Californian strains and samples

Plants showing symptoms consistent with downy mildew infection were collected during the spring of 2016 in King City, Salinas (Monterey Co.) and Davis (Yolo Co.), CA, USA. Plant hosts (S4 Table) were examined for signs of downy mildew and sporangia were isolated. Samples of sporangia were stored in sterile de-ionized water at 4°C. A phylogenetically distinct strain of *Phytophthora* (SCVWD302) was obtained from lesions taken from an unripe pear placed for five days in flooded soil and plant roots taken from underneath a California mugwort plant (*Artemisia douglasiana*). The plant was nursery-reared and then outplanted for the purposes of ecological restoration, and sampled as part of a broader survey of restoration sites in Santa Clara Co., CA. The strain produces variably-shaped, non-caducous, non-papillate sporangia (Fig 3), and has not formed oospores in culture. Sampling locations can be found in GenBank sequence accessions (S1 Table). The single living strain new to this study is *Phytophthora* taxon mugwort strain SCVWD302; the other organisms collected for this project were obligate biotrophs, with only DNA samples derived from their collection. SCVWD302 was collected with permission and supervision by Santa Clara Valley Water District on land managed by the agency. The four downy mildew samples from Yolo County, CA were collected on the campus of the University of California, Davis; the remaining samples were collected from plants in agricultural fields with permission from owners and managers who wish to remain anonymous.

Downy mildew DNA was extracted from sporangia samples using a PowerSoil kit (MO BIO, Carlsbad CA, USA) according to manufacturer's instructions. To extract genomic DNA, SCVWD302 was grown in 1 ml of clarified pea broth for 48 hours at 20 C, and mycelium was harvested with a sterilized poker. DNA was extracted using the PrepMan Ultra kit (Thermo Fisher Scientific, Waltham MA, USA) according to manufacturer's instructions. Extracted DNA was obtained from a sample of *Cyperus esculentus* infected by *Phytophthora cyperi* using the DNeasy Plant Mini Kit (QIAGEN, Venlo, Netherlands).

### DNA amplification and sequencing

Sequences from eight loci were obtained from each sample; primers and annealing temperatures for amplification and sequencing are listed in S2 Table. Methods for obtaining and analyzing the ITS sequence of *Phytophthora cyperi* will be discussed in a separate manuscript (in preparation). PCR conditions are listed in S3 Table; different amplification conditions were used for nuclear and mitochondrial loci. The FRiz forward ITS primer was initially developed for routine amplification and sequencing of *Pythium* and *Phytophthora* but proved to be useful for potentially plant-contaminated downy mildew samples due to its apparent specificity to oomycetes. In both *Bremia* species, any amplicon containing the ITS2 region produced multiple bands, including some much larger than expected, a known issue with DMPH [68]; as a result, only the ITS1 was amplified and sequenced from the two samples. The combination of BTubF4 and BTubR3 proved able to amplify the beta tubulin locus without also amplifying host genes, which was particularly problematic for the *Phytophthora cyperi* sample. The reverse primer SecYtrnC-R was used for some of the downy mildew samples that did not amplify well with SecY-R. The internal primers SecY-F2 and SecY-R2 were developed because *P*. taxon mugwort contains homopolymers in both the 5' and 3' end of its secY ORF, which rendered downstream sequences difficult to read; the additional primers listed for the cox2-cox1 contig also reflect the presence of homopolymers in many samples, both in-frame and in the cox2-cox1 intergenic spacer. PCR products were cleaned with Exo-SAP-IT (Thermo Fisher Scientific, Waltham MA, USA) according to manufacturer's instructions and Sanger sequencing performed by the UC Davis ^UC^DNA Sequencing Facility (Davis, CA, USA). Chromaseq was used to call bases and assemble contigs [69–72]; sequences were uploaded to GenBank (S1 Table).

### Species determination of downy mildew samples

Initial determinations of the downy mildew samples were based on host and sporangial morphology and then confirmed by comparison of sequence data to data from voucher specimens available in GenBank (S4 Table). For this purpose ITS rDNA and cox1 sequences were also obtained from samples. Determination of the downy mildew sample from *Pisum sativum* was made using ITS sequences in the context of work of [73]. The ITS sequence of sample RC16DM4 was distinct from all others in GenBank by 1 bp, but was most closely related to ITS sequences determined to be *P*. *viciae* s.l. in that study; it also matched a sample identified as *P*. *viciae* f. sp. *pisi* from [74] at 577/577 positions of the cox2 and 679/680 of the cox1. The sample from *Claytonia perfoliata* was determined to be *Peronospora* aff. *claytoniae* based on a 2 bp difference in ITS sequence from the *P*. *claytoniae* voucher sequenced by [15] and considering RC16DM3 was collected from a different species than the type specimen of *P*. *claytoniae* (collected from *C*. *virginica*). Although RC16DM5 differed from other accessions similarly to RC16DM2, it was clearly nested within the diversity of isolates uploaded to GenBank as *P*. *belbahrii* in all three loci compared; this could not be determined for RC16DM2 because *P*. *claytoniae* is only represented in GenBank by a single ITS accession. The sequences obtained from sample RC16DM8 differed sufficiently from the publicly available *P*. *lepidii* sequences that it was not determined to be *P*. *lepidii*. It is possible that RC16DM8 represents a previously uncharacterized lineage within a phylogenetically broad *P*. *lepidii*, as the sample was collected from *Lepidium didymum*, a host not previously sampled for genetic analysis of the species. A second species of *Perofascia* was described as this manuscript was being prepared, but sequences were not available for comparison [75].

### Compiling phylogenetic data

We used the combined nuclear and mitochondrial data set recommended by [37] from TreeBASE (M18259) to place downy mildew taxa in relation to *Phytophthora* taxa. Each of the six loci used in the current study (Table 2) was extracted from the original alignment and then manipulated individually using the program AliView [76]. We added twelve additional species of *Phytophthora* from the GenBank nucleotide collection and four species from GenBank genome-sequencing data (S1 Table). We added sequences obtained from *P*. *cyperi* and *P*. taxon mugwort for a total of 103 *Phytophthora* species. The thirteen downy mildew taxa added to the alignment were made up of eight samples collected in northern and central California and sequenced for this study, and five taxa for which genome-sequencing data was available (Table 3, S1 Table). The six loci were extracted from publicly available genome-sequencing data (S1 Table) by conducting BLAST searches of contigs of probable loci against sequence read archive accessions, ensuring contigs were properly assembled. If contigs were not available, temporary contigs were constructed from the sequence read archive data using the program MegaHit v1.0.6.1 [77]. Sequences derived from the *Plasmopara viticola* draft genome [78] were omitted from the analysis because they matched *P*. *halstedii* at the four mitochondrial loci used, suggesting the sample used for the *P*. *viticola* draft genome is chimeric in some way. In two cases, sequences from two individuals were combined into a single sample because neither had all desired loci available in GenBank (S1 Table); this was only allowed if the two strains or samples shared an identical ITS sequence. The specific names of some strains were changed to reflect recent systematic efforts. A second data set included all twenty downy mildew genera by adding seventeen taxa; all seventeen had cox2 sequences available in GenBank and sixteen also had LSU rDNA sequences available (S1 Table).

### Alignment and model/partitioning scheme selection

The LSU locus was aligned with MAFFT Q-INS-i [79,80] and the other loci were aligned with MAFFT L-INS-i [81] as amino acids. Start and stop codons were trimmed and each locus was filtered using the Phylogeny.fr Gblocks [82] webpage using the least stringent settings; the six trimmed, filtered loci were concatenated with FASconCAT [83] (Tables 1, S2 Table). The final 118-taxon alignment contained 103 *Phytophthora* taxa, thirteen downy mildew taxa and two *Pythium* s.l. outgroups; the 135-taxon alignment contained thirty downy mildew taxa (Table 1 and S1 Table). The alignments were submitted to PartitionFinder (version 2.0.0-pre13) [84,85] to simultaneously assess partitioning schemata and nucleotide models using the AICc criterion, searching 224 nucleotide models for each locus, branch length estimation linked between loci and using the “greedy” search algorithm [86] to compare potential schemata. Both alignments were partitioned as sixteen subsets (the LSU locus plus the five protein-coding loci partitioned by codon position); the best fitting model/partitioning scheme for both alignments left all subsets separate (S5 Table). To look for mito-nuclear discordance and any other incongruence between loci, a pair of trees was inferred from both the 118-taxon and 135-taxon alignments using nuclear-only and mitochondrial-only data (S2 and S3 Figs), and a single tree for each of the six loci (Table 2) from the 118-taxon alignment by itself (S4–S9 Figs).

### Saturation of sites

Saturation of sites was investigated for each of the sixteen subsets using DAMBE 6.4.28 [87]. Transition/transversion plots were first plotted against a corrected genetic distance for each of the sixteen subsets, observing that some sites did appear to be underestimating distances, and therefore may be experiencing substitution saturation [88]. The method of [89] was used to estimate the critical index of substitution saturation (I_SS.C_) value of each subset (where tree topology may begin to be inferred incorrectly) and whether I_SS.C_ significantly differed from the estimated I_SS_ of the subset (S6 Table). The five subsets corresponding to third codon positions of the protein coding loci had the highest I_SS_ values, followed by the LSU subset. For relatively symmetrical trees the ISS.c could always be significantly distinguished from the ISS, and so all sixteen subsets were included in downstream phylogenetic inference (Figs 1 and 2). Results indicated that for an extremely asymmetrical tree the five third codon position subsets had I_SS_ values that either could not be distinguished from I_SS.C_ values with p ≤ 0.001 or were greater than corresponding I_SS.C_ values (S6 Table), so, although the trees inferred in the current study were relatively symmetrical, a phylogenetic analysis was performed to observe the effect of omitting these five most saturated subsets (S1 Fig).

### Phylogenetic inference

Trees were inferred from the 118-taxon alignment using three different methods. A maximum likelihood (ML) tree was inferred using the PartitionFinder scheme (S5 Table) employing 200 best-tree searches in the program Garli 2.01 [90], with 1000 bootstrap replications providing support, added using Sumtrees.py [91]. A Bayesian likelihood consensus tree was inferred with MrBayes 3.2.6 [92], using the closest approximation of the best PartitionFinder scheme and running generations until the standard deviation of split frequencies was stably < 0.01, potential scale reduction factor values < 1.01 and runs had otherwise sufficiently converged. The GTR-CAT model of PhyloBayes 4.1c [57,93,94] with four categories of gamma-distributed rates was employed with an unpartitioned data set, generating >19,000 trees with each of the two runs and using a 5000 tree burn-in. The PhyloBayes runs successfully converged with bpcomp reporting a maxdiff of < 0.1 and meandiff of 0.002 for the consensus tree, and tracecomp reporting a maximum discrepancy < 0.08 with effective sizes > 500. To create Fig 1, the support values from the two Bayesian analyses were mapped onto the ML tree using TreeGraph2 (version 2.13) [95]. Branches were thickened if their bipartition received significant support in at least one analysis, and thickened greatly if they were significantly supported in all three analyses. Significant support was considered a bootstrap value ≥ 0.70 for ML, and a posterior probability ≥ 0.95 for Bayesian likelihood trees. Additional annotations of tree figures were performed with Inkscape 0.91 (www.inkscape.org). Fig 2 was produced with the same methods as Fig 1, omitting the PhyloBayes analysis and only thickening branches significant in both Garli and MrBayes analyses. All other phylogenetic analyses were performed with MrBayes only, including S1–S9 Figs.

## Supporting information

S1 Table List of taxa not appearing in Martin et al. [37]. DM, downy mildew; BDM, brassicolous DM; DMCC, DM with colored conidia; DMPH, DM with pyriform haustoria; GDM, graminicolous DM. Clades in parentheses are proposed (Fig 1). Strain numbers beginning with “P” refer to the World Oomycete Genetic Resource Collection. ◊Only ~800 bp of the ~1300 bp locus was available.
(DOCX)

S2 Table Oligonucleotide primers. Various combinations of primers and annealing temperatures were used for some loci; the most commonly used annealing temperature is given with a range indicated, if relevant. The most commonly used pair of primers is listed first; specific primers used to amplify and generate sequences are listed in the corresponding GenBank accessions, which are listed in S1 Table. The rps10 primers are referred to as rps10 rather than prv-9, because two different sets of primers have been published using the name prv-9 [96,97]. *Internal sequencing primer.
(DOCX)

S3 Table PCR conditions. Volumes are in μl.
(DOCX)

S4 Table List of loci used for molecular determination of Californian downy mildews. The number of matching bp is shown as a fraction of the total bp from a BLAST pairwise comparison above the matching accession. The ITS2 region was not able to be sequenced for *Bremia*, so comparisons are ITS1 only for those two species. RC16DM8 was compared against sequences of *Perofascia lepidii*.
(DOCX)

S5 Table Partitioning and model schemata selected by PartitionFinder. (DOCX)

S6 Table Results of substitution saturation tests implemented in DAMBE. I_SS_, index of substitution saturation; I_SS.C_, critical value of I_SS_; p, p-value that I_SS_ is significantly smaller than I_SS.C_; (sym), results assuming a symmetrical tree; (asym), results assuming an extremely assymetrical tree. p values ≥ 0.001 are highlighted.
(DOCX)

S1 Fig Consensus tree from MrBayes analysis of an 11-subset alignment, omitting the five most substitution-saturated subsets. The third codon-position subsets from the original alignment of 118 taxa (Fig 1) were omitted from the analysis. Branches corresponding to significantly supported bipartitions (posterior probability ≥ 0.95) are thickened. Figure created with TreeGraph2 and Inkscape.
(PDF)

S2 Fig Consensus trees from MrBayes analyses of 118-taxon nuclear-only and mitochondrial-only alignments. Branches corresponding to significantly supported bipartitions (posterior probability ≥ 0.95) are thickened. Slashes indicate branches shortened for display purposes. Putative instances of mito-nuclear discordance between subgeneric clades are highlighted. Figure created with TreeGraph2 and Inkscape.
(PDF)

S3 Fig Consensus trees from MrBayes analyses of 135-taxon nuclear-only and mitochondrial-only alignments, excerpts. Outgroups and *Phytophthora* clades 6–10 were omitted from the figure. Branches corresponding to significantly supported bipartitions (posterior probability ≥ 0.95) are thickened. The lengths of the branches connecting the common ancestors of *Eraphthora* and *Sclerophthora* were halved for display purposes. *Poakatesthia penniseti* could not be included in the nuclear-only tree. Putative instances of mito-nuclear discordance between subgeneric clades are highlighted. Figure created with TreeGraph2 and Inkscape.
(PDF)

S4 Fig Consensus tree from MrBayes analysis of LSU rDNA-only alignment. Branches corresponding to significantly supported bipartitions (posterior probability ≥ 0.95) are thickened. Scale bar units are substitutions per site. Figure created with TreeGraph2.
(PDF)

S5 Fig Consensus tree from MrBayes analysis of beta tubulin-only alignment. Branches corresponding to significantly supported bipartitions (posterior probability ≥ 0.95) are thickened. Scale bar units are substitutions per site. Figure created with TreeGraph2.
(PDF)

S6 Fig Consensus tree from MrBayes analysis of (mt) cox2-only alignment. Branches corresponding to significantly supported bipartitions (posterior probability ≥ 0.95) are thickened. Scale bar units are substitutions per site. Figure created with TreeGraph2.
(PDF)

S7 Fig Consensus tree from MrBayes analysis of (mt) nad9-only alignment. Branches corresponding to significantly supported bipartitions (posterior probability ≥ 0.95) are thickened. Scale bar units are substitutions per site. Figure created with TreeGraph2.
(PDF)

S8 Fig Consensus tree from MrBayes analysis of (mt) rps10-only alignment. Branches corresponding to significantly supported bipartitions (posterior probability ≥ 0.95) are thickened. Scale bar units are substitutions per site. Figure created with TreeGraph2.
(PDF)

S9 Fig Consensus tree from MrBayes analysis of (mt) secY-only alignment. Branches corresponding to significantly supported bipartitions (posterior probability ≥ 0.95) are thickened. Scale bar units are substitutions per site. Figure created with TreeGraph2.
(PDF)
