# Supplementary material for: Multiple origins of downy mildews and mito-nuclear discordance within the paraphyletic genus Phytophthora
Source: PLoS One. 2018 Mar 12;13(3):e0192502. doi: 10.1371/journal.pone.0192502 (PMC5846723; doi:10.1371/journal.pone.0192502)
Supplement: S1 Table — DM, downy mildew; BDM, brassicolous DM; DMCC, DM with colored conidia; DMPH, DM with pyriform haustoria; GDM, graminicolous DM. Clades in parentheses are proposed (Fig 1). Strain numbers beginning with “P” refer to the World Oomycete Genetic Resource Collection. ◊Only ~800 bp of the ~1300 bp locus was available. (DOCX) [file pone.0192502.s001.docx]

**S1 Table. List of taxa not appearing in Martin et al. (2014)**

| **A. Taxa sequenced for this study** | | | | | | | | | | | |
| --- | --- | --- | --- | --- | --- | --- | --- | --- | --- | --- | --- |
| **Genus** | **Species** | **Strain/Sample** | **Group/Clade** | **LSU** | **btub** | **cox2 + cox1** | **nad9** | | **rps10** | **secY** | **ITS** |
| *Bremia* | *lactucae* | RC16DM6 | DMPH | MF693907 | MF687228 | MF687313 | MF687321 | | MF687329 | MF687337 | MF693899 |
| *Bremia* | *sonchicola* | RC16DM7 | DMPH | MF693908 | MF687229 | MF687314 | MF687322 | | MF687330 | MF687338 | MF693900 |
| *Perofascia* | sp. | RC16DM8 | BDM | MF693909 | MF687230 | MF687315 | MF687323 | | MF687331 | MF687339 | MF693901 |
| *Peronospora* | *belbahrii* | RC16DM5 | DMCC | MF693906 | MF687227 | MF687312 | MF687320 | | MF687328 | MF687336 | MF693898 |
| *Peronospora* | aff. *claytoniae* | RC16DM3 | DMCC | MF693904 | MF687225 | MF687310 | MF687318 | | MF687326 | MF687334 | MF693896 |
| *Peronospora* | *effusa* | RC16DM1 | DMCC | MF693902 | MF687223 | MF687308 | MF687316 | | MF687324 | MF687332 | MF693894 |
| *Peronospora* | *schactii* | RC16DM2 | DMCC | MF693903 | MF687224 | MF687309 | MF687317 | | MF687325 | MF687333 | MF693895 |
| *Peronospora* | *viciae*s.l. | RC16DM4 | DMCC | MF693905 | MF687226 | MF687311 | MF687319 | | MF687327 | MF687335 | MF693897 |
| *Phytophthora* | *cyperi* | MVAP06098582 | (14) | KY587789 | KY608800 | KC136846 | KC151259 | | KC151260 | KY608801 |  |
| *Phytophthora* | taxon mugwort | SCVWD302 | (13) | MF693892 | MF687222 | MF687218 | MF687220 | | MF687219 | MF687221 | MF693893 |
| **B. Taxa added from GenBank genome-sequencing data**  DM, downy mildew; BDM, brassicolous DM; DMCC, DM with colored conidia; DMPH, DM with pyriform haustoria; GDM, graminicolous DM. Clades in parentheses are proposed (Fig 1). Strain numbers beginning with “P” refer to the World Oomycete Genetic Resource Collection. ◊Only ~800 bp of the ~1300 bp locus was available. | | | | | | | | | | | |
| **Genus** | **Species** | **Strain/Sample** | **Group/Clade** | **BioProject** |  | | | | | | |
| *Hyaloperonospora* | *arabidopsidis* | Noks1 | BDM | PRJNA298674 |  |  |  |  |  |  |  |
| *Peronospora* | *tabacina* | 968-J2 | DMCC | PRJEB6937 |  |  |  |  |  |  |  |
| *Phytophthora* | *agathidicida* | NZFS 3770 | 5 | PRJNA290659 |  |  |  |  |  |  |  |
| *Phytophthora* | *litchii* | SHS3 | 4 | PRJNA290406 |  |  |  |  |  |  |  |
| *Phytophthora* | *pluvialis* | LC9-1 | 3 | PRJNA290686 |  |  |  |  |  |  |  |
| *Phytophthora* | taxon totara | NZFS 3727 | (15) | PRJNA290833 |  |  |  |  |  |  |  |
| *Plasmopara* | *halstedii* | (not specified) | DMPH | PRJEB6932 |  |  |  |  |  |  |  |
| *Pseudoperonospora* | *cubensis* | MSU1 | DMCC | PRJNA80635 |  |  |  |  |  |  |  |
| *Sclerospora* | *graminicola* | UoM-SG-Pathotype1 | GDM | PRJNA325098 |  |  |  |  |  |  |  |
| **C. Downy mildew taxa added from the GenBank nucleotide collection (partial coverage)** | | | | | | | | | | | |
| **Genus** | **Species** | **Sample** | **Group** | **LSU** | **btub** | **cox2** |  | | | | |
| *Baobabopsis* | *donbarrettii* | BRIP54675 | GDM | KT248945 |  | KT248948 |  |  |  |  |  |
| *Basidiophora* | *entospora* | HV 123 | DMPH | AY035513 |  | DQ365699 |  |  |  |  |  |
| *Benua* | *kellermanii* | HV 2071 | DMPH | DQ361226 |  | DQ365700 |  |  |  |  |  |
| *Eraphthora* | *butleri* | FR-0046004 | GDM | KT248944 |  | KP965746 |  |  |  |  |  |
| *Graminivora* | *graminicola* | AR327 | GDM | DQ195167 |  | DQ365702 |  |  |  |  |  |
| *Novotelnova* | *scorzonerae* | HV 2165 | DMPH | KT249532 |  | KJ654164 |  |  |  |  |  |
| *Paraperonospora* | *leptosperma* | HV 383 | DMPH | AY035515 |  | DQ365712 |  |  |  |  |  |
| *Paraperonospora* | *tanaceti* | GLM73325 | DMPH | KT249495 |  | KJ654165 |  |  |  |  |  |
| *Peronosclerospora* | *eriochloae* | FR-0046005 | GDM | HQ261786 |  | HQ261813 |  |  |  |  |  |
| *Peronosclerospora* | *sacchari* | BRIP44241a | GDM | HQ261764 |  | HQ261791 |  |  |  |  |  |
| *Plasmopara* | *obducens* | HV 306 | DMPH | AY250181 | DQ361156 | DQ365757 |  |  |  |  |  |
| *Plasmoverna* | *anemones-ranunculoides* | HUH583 | DMPH | GU361037 |  | KJ654171 |  |  |  |  |  |
| *Plasmoverna* | *pygmaea* | AR86^/MG1846* | DMPH | AF119605^ | DQ361160^ | DQ365761* |  |  |  |  |  |
| *Poakatesthia* | *penniseti* | IMI 137328c | GDM |  |  | EF426475 |  |  |  |  |  |
| *Protobremia* | *sphaerosperma* | ProtR112010 | DMPH | KT249533 |  | KJ654172 |  |  |  |  |  |
| *Sclerophthora* | *macrospora* | HUH892 | GDM | EU826119 |  | KP965748 |  |  |  |  |  |
| *Viennotia* | *oplismeni* | HV 11 | GDM | AY035527 | DQ361168 | DQ365769 |  |  |  |  |  |
| **D. *Phytophthora* taxa added from the GenBank nucleotide collection** | | | | | | | |  |  |  |  |
| **Genus** | **Species** | **Strain** | **Clade** | **LSU** | **btub** | **cox2 + cox1** | **nad9** | | **rps10** | **secY** |  |
| *Phytophthora* | *austrocedri* | P15132 | 8 | JF771634 | HQ917873 | HQ917881 | HQ917857 | | JF770870 | JF770495 |  |
| *Phytophthora* | *chlamydospora* | P6132^/P10669* | 6 | EU080123^ | EU080119^ | JF771550* | JF771986* | | JF771181* | JF770806* |  |
| *Phytophthora* | *gallica* | P16826 | 10 | GU594853 | HM534947 | HM534964 | JQ439050 | | JQ439194 | JQ439321 |  |
| *Phytophthora* | *hydropathica* | P16857 | 9 | GU59484◊ | HM534942 | HM534959 | JQ439063 | | JQ439206 | JQ439331 |  |
| *Phytophthora* | *irrigata* | P16861 | 9 | GU594852◊ | HM534946 | HM534963 | JQ439066 | | JQ439209 | JQ439334 |  |
| *Phytophthora* | *parsiana* | P15164 | 9 | GU594863◊ | HM534936 | HM535000 | JQ439123 | | JQ439268 | JQ439390 |  |
| *Phytophthora* | *richardiae* | P6875 | 9 | EU080548 | EU080545 | GU222131 | JQ439151 | | JQ439278 | JF770839 |  |
| *Phytophthora* | sp. aff. *chicorii* | P6207 | 8 | EU079885 | EU079881 | GU222113 | JF771997 | | JF771190 | JF770815 |  |
| *Phytophthora* | sp. *canalensis* | P10456 | 6 | EU079574 | EU079570 | GU221956 | JF771656 | | JF770889 | JF770512 |  |
| *Phytophthora* | sp. *napoensis* | P8221 | 9 | GU594882◊ | HM534931 | HM534995 | JF771954 | | JQ439261 | JQ439383 |  |
| *Phytophthora* | sp. *novaeguineae* | P1256 | 5 | JF273228◊ | HM991922 | GU222095 | JF771973 | | JF771168 | JF770793 |  |
| *Phytophthora* | sp. *thermophilum* | P10457 | 9 | GU594856◊ | HM534915 | HM534979 | JF772044 | | JF771228 | JF770852 |  |
