# Supplementary material for: Multiple origins of downy mildews and mito-nuclear discordance within the paraphyletic genus Phytophthora
Source: PLoS One. 2018 Mar 12;13(3):e0192502. doi: 10.1371/journal.pone.0192502 (PMC5846723; doi:10.1371/journal.pone.0192502)
Supplement: S2 Table — Various combinations of primers and annealing temperatures were used for some loci; the most commonly used annealing temperature is given with a range indicated, if relevant. The most commonly used pair of primers is listed first; specific primers used to amplify and generate sequences are listed in the corresponding GenBank accessions, which are listed in S1 Table. The rps10 primers are referred to as rps10 rather than prv-9, because two different sets of primers have been published using the name prv-9 [96,97]. *Internal sequencing primer. (DOCX) [file pone.0192502.s002.docx]

|  | Locus | Anneal |  | Primer sequences 5'-3' | Reference |
| --- | --- | --- | --- | --- | --- |
| Nuclear | ITS rDNA | 57 | ▶  **◀**  ◀ | FRiz CTCGCCATTTAGAGGAAGGTGAA  ITS4TT TTCCTCCGCTTATTGATATGCTT  B58S3 GAGATCCRTTGYTRAAAGTT | This study  This study  Bokulich & Mills (2013) [98] |
|  | LSU rDNA | 55 | ▶  ◀  ◀ | LR0R ACCCGCTGAACTTAAGC  Un-Lo28S1220 GTTGTTACACACTCCTTAGCGGAT  Un-Lo28S576B^a^ CTCCTTGGTCCGTGTTTCAAGACG | Cubeta et al. (1991) [99]  Bala et al. (2010) [100]  Bakkeren et al. (2000) [101] |
|  | beta-tubulin  (btub) | 59  (57-64) | ▶  ◀  ▶  ▶  ◀ | BTubF4 GCTYGTTCACATYCAGGGT  BTubR3 CRTCCTCRTCGAACTCGCCCTC  BTubF1 GCCAAGTTCTGGGAGGTCATC  BTubF1A GCCAAGTTCTGGGARGTSAT  BTubR1 CCTGGTACTGCTGGTACTCAG | This study  This study  Blair et al. (2008) [36]  Blair et al. (2008) [36]  Kroon et al. (2004) [61] |
| Mitochondrial | cox2  +spacer | 54-55 | ▶  ▶  ◀  ◀  ◀ | FM75 CCTTGGCAATTAGGATTTCAAGAT  FM35 CAGAACCTTGGCAATTAGG  CoxFRizA TTTGATTACCTGGTTGTGCTAATTC  FM78^a^ ACAAATTTCACTACATTGTCC  FMPh-10b GCAAAAGCACTAAAAATTAAATATAA | Martin & Tooley (2003) [102]  Martin (2000) [103]  This study  Martin & Tooley (2003) [102]  Martin et al. (2004) [104] |
|  | cox1 | 55 | ▶  ◀  ▶  ▶  ▶  ◀ | CoxFrizB AATGAATTTTCAAAATATAAATAAATGG  FM83 CTCCAATAAAAAATAACCAAAAATG  CoxFRizC ATCTGATGTYTTACATTCWTGGGC  OomCox1-Levup TCAWCWMGATGGCTTTTTTCAAC  FM84 TTTAATTTTTAGTGCTTTTGC  FM85 AACTTGACTAATAATACCAAA | This study  Martin & Tooley (2003) [102]  This study  Robideau et al. (2011)  Martin & Tooley (2003) [102]  Martin & Tooley (2003) [102] |
|  | nad9 | 61 | ▶  ◀ | nad9-F TACAACAAGAATTAATGAGAAC  nad9-R TTAAAATTTGTACTACTAACAT | Martin & Coffey (2012) [97]  Martin & Coffey (2012) [97] |
|  | rps10 | 60 | ▶  ◀ | rps10-F GTATACTCTAACCAACTGAGT  rps10-R GTTGGTTAGAGTAAAAGACT | Martin & Coffey (2012) [97]  Martin & Coffey (2012) [97] |
|  | secY | 61  (57-63) | ▶  ◀  ◀  ▶  ◀ | SecY-F TCTATCGTGTTTACCAATTTC  SecY-R TAACAAATGGATCTTCTTTAAAA  SecYtrnC-R CATTACCATTATGTTATCTAGCC  SecY-F2^a^ TGTTAAAATATTTTATYTTTACAAATATTACTG  SecY-R2^a^ AATAAAATCAAAATAATTATTAATTTTHGGTTCAA | Martin (2008) [96]  Martin (2008) [96]  This study  This study  This study |

**S2 Table. Oligonucleotide primers**

Various combinations of primers and annealing temperatures were used for some loci; the most commonly used annealing temperature is given with a range indicated, if relevant. The most commonly used pair of primers is listed first; specific primers used to amplify and generate sequences are listed in the corresponding GenBank accessions, which are listed in S1 Table. The rps10 primers are referred to as rps10 rather than prv-9, because two different sets of primers have been published using the name prv-9 (Martin 2008 [96], Martin and Coffey 2012 [97]). *Internal sequencing primer.
