# Supplementary material for: Multiple origins of downy mildews and mito-nuclear discordance within the paraphyletic genus Phytophthora
Source: PLoS One. 2018 Mar 12;13(3):e0192502. doi: 10.1371/journal.pone.0192502 (PMC5846723; doi:10.1371/journal.pone.0192502)
Supplement: S3 Table — Volumes are in μl. (DOCX) [file pone.0192502.s003.docx]

**S3 Table. PCR conditions**

| **PCR ingredients** | | | **Nuclear** | **Mitochondrial** |
| --- | --- | --- | --- | --- |
| AmpliTaq buffer (10X) | | | 2.5 | 2.5 |
| MgCl_2_ (25mM) | | | 1.5 (2.5) | 3.0 (4.0) |
| dNTPs (10 mM each) | | | 0.5 | 0.25 |
| Bovine Serum Albumin (20 ug/μl) | | | 0.1 | 0 |
| AmpliTaq Taq (5 U/μl) | | | 0.1 | 0.2 |
| forward primer (10 μM) | | | 0.5 | 1.25 |
| reverse primer (10 μM) | | | 0.5 | 1.25 |
| molecular grade H_2_0 | | | 16.9 (15.9) | 16.05 (15.05) |
| DNA template (~20 ng/μl) | | | 2 | 0.5 |
| (Reaction total) | | | 25 | 25 |
|  | | | | |
| **Cycling conditions** | | | **< 1000 bp** | ≥ **1000 bp** |
| Denature | 94°C | 1X | 4 min | 4 min |
| Denature | 94°C | 35X | 30 sec | 30 sec |
| Anneal | 54-63°C |  | 30 sec | 30 sec |
| Extension | 72°C |  | 30 sec | 1 min |
| Extension | 72°C | 1X | 10 min | 10 min |

Volumes are in μl
