# Supplementary material for: Multiple origins of downy mildews and mito-nuclear discordance within the paraphyletic genus Phytophthora
Source: PLoS One. 2018 Mar 12;13(3):e0192502. doi: 10.1371/journal.pone.0192502 (PMC5846723; doi:10.1371/journal.pone.0192502)
Supplement: S4 Table — The number of matching bp is shown as a fraction of the total bp from a BLAST pairwise comparison above the matching accession. The ITS2 region was not able to be sequenced for Bremia, so comparisons are ITS1 only for those two species. RC16DM8 was compared against sequences of Perofascia lepidii. (DOCX) [file pone.0192502.s004.docx]

##### **S4 Table. List of loci used for molecular determination of Californian downy mildews**

| **Sample** | **Host** | **Species** | **ITS** | **LSU** | **cox2** | **cox1** | **rps10** | **%ID** |
| --- | --- | --- | --- | --- | --- | --- | --- | --- |
| RC16 DM1 | ***Spinacia oleracea*** | ***Peronospora effusa*** | 797/798 KP330843 |  | 1598/1598 KP330671 | | 519/519 KP331023 | 99.97 |
| RC16 DM2 | ***Beta***  ***vulgaris*** | ***Peronospora schactii*** | 798/798 KP330786 |  | 1635/1635 KP330610 | | 519/519 KP330966 | 100.0 |
| RC16 DM3 | ***Claytonia perfoliata*** | ***Peronospora***  **aff. *claytoniae*** | 795/797 AY198281 |  |  |  |  | 99.75 |
| RC16 DM4 | ***Pisum***  ***sativum*** | ***Peronospora viciae* s.l.** | 844/845 EF174889 |  | 577/577 KJ654233 | 679/680 KJ654084 |  | 99.90 |
| RC16 DM5 | ***Ocimum basilicum*** | ***Peronospora belbahrii*** | 854/856 KJ960193 |  | 573/577 KJ654229 | 679/680 KJ654080 | **nad9** | 99.67 |
| RC16 DM6 | ***Lactuca***  ***sativa*** | ***Bremia***  ***lactucae*** | (258/258)  KT249021 | 1229/1229 KT249411 | 539/539 KP684734 | 629/629 KP684534 | 470/470 FJ810101 | 100.0 |
| RC16 DM7 | ***Sonchus oleraceus*** | ***Bremia sonchicola*** | (257/257)  KT249072 | 1229/1229 KT249462 | 539/539  KP684853 | 629/629 KP684657 | **btub** | 100.0 |
| RC16 DM8 | ***Lepidium didymum*** | ***Perofascia***  **sp.** | 745/796 AY211014 | 977/1013 DQ361228 | 532/579 KJ654166 |  | 689/716 DQ361114 | 94.81 |

##### The number of matching bp is shown as a fraction of the total bp from a BLAST pairwise comparison above the matching accession. The ITS2 region was not able to be sequenced for *Bremia,* so comparisons are ITS1 only for those two species. RC16DM8 was compared against sequences of *Perofascia lepidii*.
