# Supplementary material for: Multiple origins of downy mildews and mito-nuclear discordance within the paraphyletic genus Phytophthora
Source: PLoS One. 2018 Mar 12;13(3):e0192502. doi: 10.1371/journal.pone.0192502 (PMC5846723; doi:10.1371/journal.pone.0192502)
Supplement: S5 Table — (DOCX) [file pone.0192502.s005.docx]

**S5 Table. Partitioning and model schemata selected by PartitionFinder**

| **Subset#** | **Locus** | **bp** | **Model (118 taxa)** | **Model (135 taxa)** |
| --- | --- | --- | --- | --- |
| 1 | lsu | 1299 | GTR+I+G | GTR+I+G |
| 2 | btub_pos1 | 379 | GTR+I+G | GTR+I+G |
| 3 | btub_pos2 | 379 | TVM+I | TVM |
| 4 | btub_pos3 | 379 | SYM+I+G | SYM+I+G |
| 5 | cox2_pos1 | 226 | TVM+I+G | TVM+I+G |
| 6 | cox2_pos2 | 226 | TRN+I+G | TRN+I+G |
| 7 | cox2_pos3 | 226 | GTR+G | GTR+I+G |
| 8 | nad9_pos1 | 184 | TVM+I+G | TVM+I+G |
| 9 | nad9_pos2 | 184 | GTR+I+G | GTR+I+G |
| 10 | nad9_pos3 | 184 | GTR+I+G | GTR+I+G |
| 11 | rps10_pos1 | 107 | TIM+I+G | TIM+I+G |
| 12 | rps10_pos2 | 107 | GTR+G | GTR+G |
| 13 | rps10_pos3 | 107 | TVM+G | TVM+G |
| 14 | secY_pos1 | 243 | TVM+I+G | TVM+I+G |
| 15 | secY_pos2 | 243 | GTR+I+G | GTR+I+G |
| 16 | secY_pos3 | 243 | GTR+I+G | GTR+I+G |
