# Supplementary material for: Multiple origins of downy mildews and mito-nuclear discordance within the paraphyletic genus Phytophthora
Source: PLoS One. 2018 Mar 12;13(3):e0192502. doi: 10.1371/journal.pone.0192502 (PMC5846723; doi:10.1371/journal.pone.0192502)
Supplement: S6 Table — ISS, index of substitution saturation; ISS.C, critical value of ISS; p, p-value that ISS is significantly smaller than ISS.C; (sym), results assuming a symmetrical tree; (asym), results assuming an extremely assymetrical tree. p values ≥ 0.001 are highlighted. (DOCX) [file pone.0192502.s006.docx]

**S6 Table. Results of substitution saturation tests implemented in DAMBE**

| Subset | # OTU | I_SS_ | I_SS.C_ (sym) | p  (sym) | I_SS.C_ (asy) | p  (asy) |
| --- | --- | --- | --- | --- | --- | --- |
| lsu | 4 | 0.298 | 0.829 | 0.0000 | 0.798 | 0.0000 |
| lsu | 8 | 0.271 | 0.802 | 0.0000 | 0.700 | 0.0000 |
| lsu | 16 | 0.259 | 0.785 | 0.0000 | 0.597 | 0.0000 |
| lsu | 32 | 0.253 | 0.766 | 0.0000 | 0.475 | 0.0000 |
| btub_pos1 | 4 | 0.140 | 0.787 | 0.0000 | 0.756 | 0.0000 |
| btub_pos1 | 8 | 0.132 | 0.740 | 0.0000 | 0.629 | 0.0000 |
| btub_pos1 | 16 | 0.136 | 0.698 | 0.0000 | 0.489 | 0.0000 |
| btub_pos1 | 32 | 0.137 | 0.689 | 0.0000 | 0.359 | 0.0000 |
| btub_pos2 | 4 | 0.105 | 0.787 | 0.0000 | 0.756 | 0.0000 |
| btub_pos2 | 8 | 0.094 | 0.740 | 0.0000 | 0.629 | 0.0000 |
| btub_pos2 | 16 | 0.098 | 0.698 | 0.0000 | 0.489 | 0.0000 |
| btub_pos2 | 32 | 0.096 | 0.689 | 0.0000 | 0.359 | 0.0000 |
| btub_pos3 | 4 | 0.356 | 0.787 | 0.0000 | 0.756 | 0.0000 |
| btub_pos3 | 8 | 0.359 | 0.740 | 0.0000 | 0.629 | 0.0000 |
| btub_pos3 | 16 | 0.366 | 0.698 | 0.0000 | 0.489 | 0.0000 |
| btub_pos3 | 32 | 0.373 | 0.689 | 0.0000 | 0.359 | 0.5458 |
| cox2_pos1 | 4 | 0.127 | 0.777 | 0.0000 | 0.762 | 0.0000 |
| cox2_pos1 | 8 | 0.128 | 0.732 | 0.0000 | 0.631 | 0.0000 |
| cox2_pos1 | 16 | 0.133 | 0.653 | 0.0000 | 0.458 | 0.0000 |
| cox2_pos1 | 32 | 0.138 | 0.685 | 0.0000 | 0.364 | 0.0000 |
| cox2_pos2 | 4 | 0.124 | 0.777 | 0.0000 | 0.762 | 0.0000 |
| cox2_pos2 | 8 | 0.124 | 0.732 | 0.0000 | 0.631 | 0.0000 |
| cox2_pos2 | 16 | 0.130 | 0.653 | 0.0000 | 0.458 | 0.0000 |
| cox2_pos2 | 32 | 0.135 | 0.685 | 0.0000 | 0.364 | 0.0003 |
| cox2_pos3 | 4 | 0.340 | 0.777 | 0.0000 | 0.762 | 0.0000 |
| cox2_pos3 | 8 | 0.349 | 0.732 | 0.0000 | 0.631 | 0.0000 |
| cox2_pos3 | 16 | 0.357 | 0.653 | 0.0000 | 0.458 | 0.0014 |
| cox2_pos3 | 32 | 0.366 | 0.685 | 0.0000 | 0.364 | 0.9573 |
| nad9_pos1 | 4 | 0.154 | 0.777 | 0.0000 | 0.773 | 0.0000 |
| nad9_pos1 | 8 | 0.154 | 0.737 | 0.0000 | 0.643 | 0.0000 |
| nad9_pos1 | 16 | 0.159 | 0.635 | 0.0000 | 0.454 | 0.0000 |
| nad9_pos1 | 32 | 0.164 | 0.697 | 0.0000 | 0.388 | 0.0000 |
| nad9_pos2 | 4 | 0.091 | 0.777 | 0.0000 | 0.773 | 0.0000 |
| nad9_pos2 | 8 | 0.092 | 0.737 | 0.0000 | 0.643 | 0.0000 |
| nad9_pos2 | 16 | 0.098 | 0.635 | 0.0000 | 0.454 | 0.0000 |
| nad9_pos2 | 32 | 0.101 | 0.697 | 0.0000 | 0.388 | 0.0000 |
| nad9_pos3 | 4 | 0.278 | 0.777 | 0.0000 | 0.773 | 0.0000 |
| nad9_pos3 | 8 | 0.291 | 0.737 | 0.0000 | 0.643 | 0.0000 |
| nad9_pos3 | 16 | 0.303 | 0.635 | 0.0000 | 0.454 | 0.0000 |
| nad9_pos3 | 32 | 0.311 | 0.697 | 0.0000 | 0.388 | 0.0086 |
| rps10_pos1 | 4 | 0.212 | 0.800 | 0.0000 | 0.836 | 0.0000 |
| rps10_pos1 | 8 | 0.219 | 0.786 | 0.0000 | 0.728 | 0.0000 |
| rps10_pos1 | 16 | 0.227 | 0.592 | 0.0000 | 0.477 | 0.0000 |
| rps10_pos1 | 32 | 0.238 | 0.785 | 0.0000 | 0.552 | 0.0000 |
| rps10_pos2 | 4 | 0.102 | 0.800 | 0.0000 | 0.836 | 0.0000 |
| rps10_pos2 | 8 | 0.106 | 0.786 | 0.0000 | 0.728 | 0.0000 |
| rps10_pos2 | 16 | 0.111 | 0.592 | 0.0000 | 0.477 | 0.0000 |
| rps10_pos2 | 32 | 0.118 | 0.785 | 0.0000 | 0.552 | 0.0000 |
| rps10_pos3 | 4 | 0.340 | 0.800 | 0.0000 | 0.836 | 0.0000 |
| rps10_pos3 | 8 | 0.363 | 0.786 | 0.0000 | 0.728 | 0.0000 |
| rps10_pos3 | 16 | 0.383 | 0.592 | 0.0000 | 0.477 | 0.0387 |
| rps10_pos3 | 32 | 0.398 | 0.785 | 0.0000 | 0.552 | 0.0008 |
| secY_pos1 | 4 | 0.193 | 0.777 | 0.0000 | 0.760 | 0.0000 |
| secY_pos1 | 8 | 0.198 | 0.732 | 0.0000 | 0.629 | 0.0000 |
| secY_pos1 | 16 | 0.203 | 0.660 | 0.0000 | 0.461 | 0.0000 |
| secY_pos1 | 32 | 0.209 | 0.683 | 0.0000 | 0.359 | 0.0000 |
| secY_pos2 | 4 | 0.155 | 0.777 | 0.0000 | 0.760 | 0.0000 |
| secY_pos2 | 8 | 0.162 | 0.732 | 0.0000 | 0.629 | 0.0000 |
| secY_pos2 | 16 | 0.170 | 0.660 | 0.0000 | 0.461 | 0.0000 |
| secY_pos2 | 32 | 0.177 | 0.683 | 0.0000 | 0.359 | 0.0000 |
| secY_pos3 | 4 | 0.331 | 0.777 | 0.0000 | 0.760 | 0.0000 |
| secY_pos3 | 8 | 0.348 | 0.732 | 0.0000 | 0.629 | 0.0000 |
| secY_pos3 | 16 | 0.362 | 0.660 | 0.0000 | 0.461 | 0.0002 |
| secY_pos3 | 32 | 0.373 | 0.683 | 0.0000 | 0.359 | 0.5879 |

I_SS_, index of substitution saturation; I_SS.C_, critical value of I_SS_; p, p-value that I_SS_ is significantly smaller than I_SS.C_; (sym), results assuming a symmetrical tree; (asym), results assuming an extremely assymetrical tree. p values ≥ 0.001 are highlighted.
