# Supplementary material for: Multiple origins of downy mildews and mito-nuclear discordance within the paraphyletic genus Phytophthora
Source: PLoS One. 2018 Mar 12;13(3):e0192502. doi: 10.1371/journal.pone.0192502 (PMC5846723; doi:10.1371/journal.pone.0192502)
Supplement: S3 Fig — Outgroups and Phytophthora clades 6–10 were omitted from the figure. Branches corresponding to significantly supported bipartitions (posterior probability ≥ 0.95) are thickened. The lengths of the branches connecting the common ancestors of Eraphthora and Sclerophthora were halved for display purposes. Poakatesthia penniseti could not be included in the nuclear-only tree. Putative instances of mito-nuclear discordance between subgeneric clades are highlighted. Figure created with TreeGraph2 and Inkscape. (PDF) [file pone.0192502.s009.pdf]

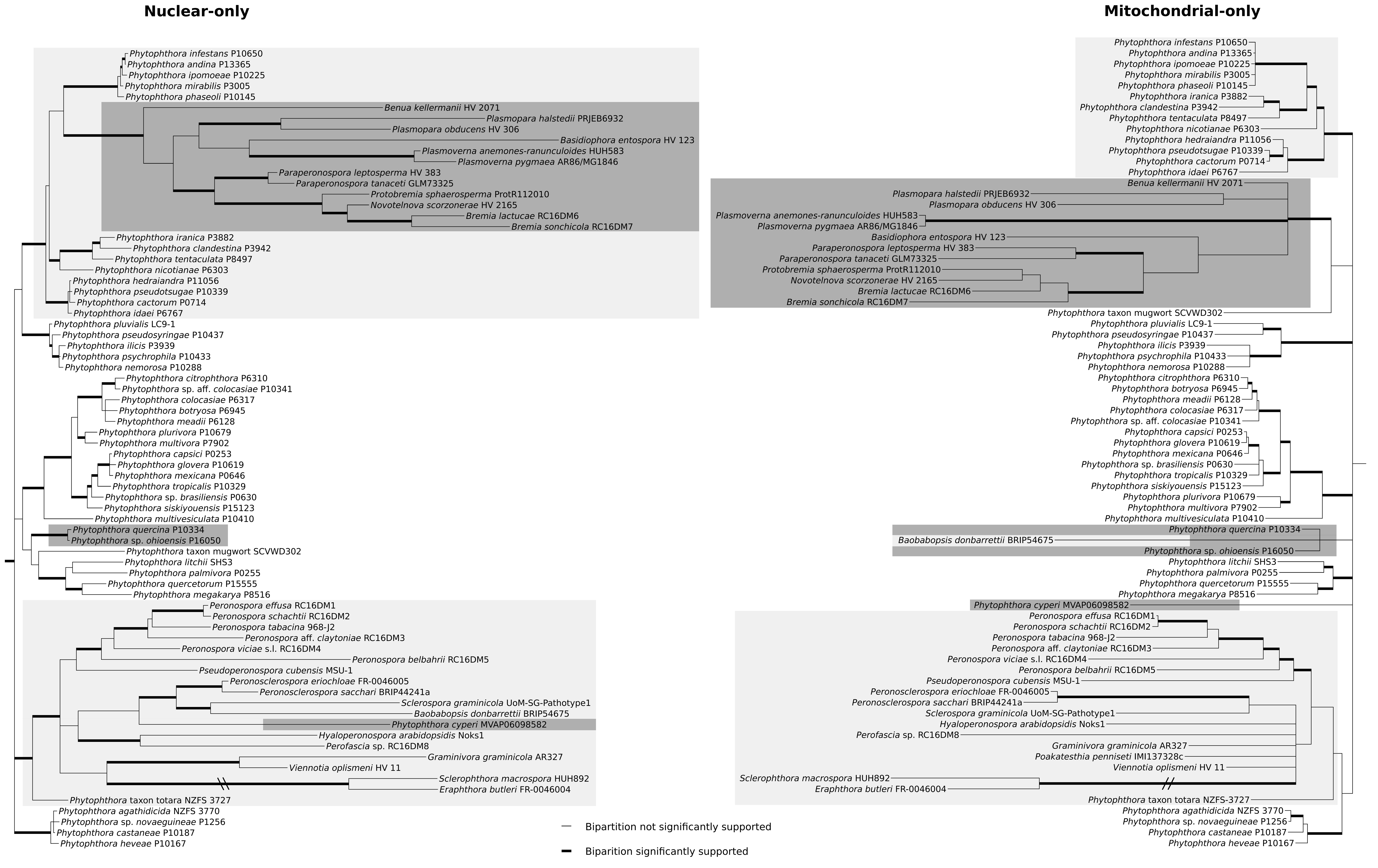
Nuclear-only
Mitochondrial-only
Phytophthora infestans P10650
Phytophthora andina P13365
Phytophthora ipomoeae P10225
Phytophthora mirabilis P3005
Phytophthora phaseoli P10145
Benua kellermanii HV 2071
Plasmopara halstedii PRJEB6932
Plasmopara obducens HV 306
Basidiophora entospora HV 123
Plasmoverna anemones-ranunculoides HUH583
Plasmoverna pygmaea AR86/MG1846
Paraperonospora leptosperma HV 383
Paraperonospora tanaceti GLM73325
Protobremia sphaerosperma ProtR112010
Novotelnova scorzonerae HV 2165
Bremia lactucae RC16DM6
Bremia sonchicola RC16DM7
Phytophthora iranica P3882
Phytophthora clandestina P3942
Phytophthora tentaculata P8497
Phytophthora nicotianae P6303
Phytophthora hedraiandra P11056
Phytophthora pseudotsugae P10339
Phytophthora cactorum P0714
Phytophthora idaei P6767
Phytophthora pluvialis LC9-1
Phytophthora pseudosyringae P10437
Phytophthora ilicis P3939
Phytophthora psychrophila P10433
Phytophthora nemorosa P10288
Phytophthora citrophthora P6310
Phytophthora sp. aff. colocasiae P10341
Phytophthora colocasiae P6317
Phytophthora botryosa P6945
Phytophthora meadii P6128
Phytophthora plurivora P10679
Phytophthora multivora P7902
Phytophthora capsici P0253
Phytophthora glovera P10619
Phytophthora mexicana P0646
Phytophthora tropicalis P10329
Phytophthora sp. brasiliensis P0630
Phytophthora siskiyouensis P15123
Phytophthora multivesiculata P10410
Phytophthora quercina P10334
Phytophthora sp. ohioensis P16050
Phytophthora taxon mugwort SCVWD302
Phytophthora litchii SHS3
Phytophthora palmivora P0255
Phytophthora quercetorum P15555
Phytophthora megakarya P8516
Peronospora effusa RC16DM1
Peronospora schachtii RC16DM2
Peronospora tabacina 968-J2
Peronospora aff. claytoniae RC16DM3
Peronospora viciae s.l. RC16DM4
Peronospora belbahrii RC16DM5
Pseudoperonospora cubensis MSU-1
Peronosclerospora eriochloae FR-0046005
Peronosclerospora sacchari BRIP44241a
Sclerospora graminicola UoM-SG-Pathotype1
Baobabopsis donbarrettii BRIP54675
Phytophthora cyperi MVAP06098582
Hyaloperonospora arabidopsidis Noks1
Perofascia sp. RC16DM8
Graminivora graminicola AR327
Viennotia oplismeni HV 11
Sclerophthora macrospora HUH892
Eraphthora butleri FR-0046004
Phytophthora taxon totara NZFS 3727
Phytophthora agathidicida NZFS 3770
Phytophthora sp. novaeguineae P1256
Phytophthora castaneae P10187
Phytophthora heveae P10167
Poakatesthia penniseti IMI137328c
Phytophthora taxon totara NZFS-3727
Bipartition not significantly supported
Biparition significantly supported
