# Supplementary material for: Multiple origins of downy mildews and mito-nuclear discordance within the paraphyletic genus Phytophthora
Source: PLoS One. 2018 Mar 12;13(3):e0192502. doi: 10.1371/journal.pone.0192502 (PMC5846723; doi:10.1371/journal.pone.0192502)
Supplement: S5 Fig — Branches corresponding to significantly supported bipartitions (posterior probability ≥ 0.95) are thickened. Scale bar units are substitutions per site. Figure created with TreeGraph2. (PDF) [file pone.0192502.s011.pdf]

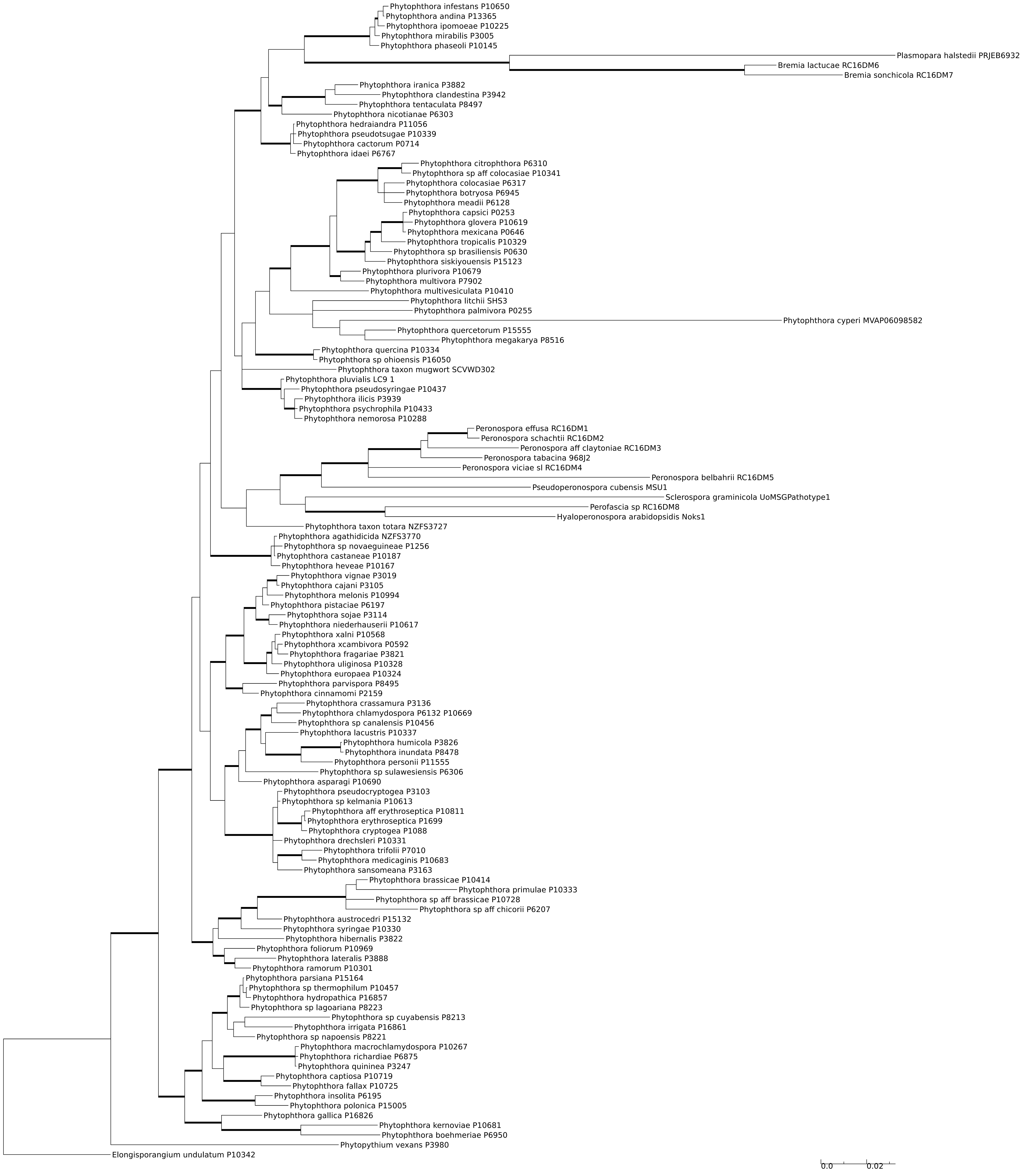
Phytophthora infestans P10650
Phytophthora andina P13365
Phytophthora ipomoeae P10225
Phytophthora mirabilis P3005
Phytophthora phaseoli P10145
Plasmopara halstedii PRJEB6932
Bremia lactucae RC16DM6
Bremia sonchicola RC16DM7
Phytophthora iranica P3882
Phytophthora clandestina P3942
Phytophthora tentaculata P8497
Phytophthora nicotianae P6303
Phytophthora hedraiandra P11056
Phytophthora pseudotsugae P10339
Phytophthora cactorum P0714
Phytophthora idaei P6767
Phytophthora citrophthora P6310
Phytophthora sp aff colocasiae P10341
Phytophthora colocasiae P6317
Phytophthora botryosa P6945
Phytophthora meadii P6128
Phytophthora capsici P0253
Phytophthora glovera P10619
Phytophthora mexicana P0646
Phytophthora tropicalis P10329
Phytophthora sp brasiliensis P0630
Phytophthora siskiyouensis P15123
Phytophthora plurivora P10679
Phytophthora multivora P7902
Phytophthora multivesiculata P10410
Phytophthora litchii SHS3
Phytophthora palmivora P0255
Phytophthora cyperi MVAP06098582
Phytophthora quercetorum P15555
Phytophthora megakarya P8516
Phytophthora quercina P10334
Phytophthora sp ohioensis P16050
Phytophthora taxon mugwort SCVWD302
Phytophthora pluvialis LC9 1
Phytophthora pseudosyringae P10437
Phytophthora ilicis P3939
Phytophthora psychrophila P10433
Phytophthora nemorosa P10288
Peronospora effusa RC16DM1
Peronospora schachtii RC16DM2
Peronospora aff claytoniae RC16DM3
Peronospora tabacina 968J2
Peronospora viciae sl RC16DM4
Peronospora belbahrii RC16DM5
Pseudoperonospora cubensis MSU1
Sclerospora graminicola UoMSGPathotype1
Perofascia sp RC16DM8
Hyaloperonospora arabidopsidis Noks1
Phytophthora taxon totara NZFS3727
Phytophthora agathidicida NZFS3770
Phytophthora sp novaeguineae P1256
Phytophthora castaneae P10187
Phytophthora heveae P10167
Phytophthora vignae P3019
Phytophthora cajani P3105
Phytophthora melonis P10994
Phytophthora pistaciae P6197
Phytophthora sojae P3114
Phytophthora niederhauserii P10617
Phytophthora xalni P10568
Phytophthora xcambivora P0592
Phytophthora fragariae P3821
Phytophthora uliginosa P10328
Phytophthora europaea P10324
Phytophthora parvispora P8495
Phytophthora cinnamomi P2159
Phytophthora crassamura P3136
Phytophthora chlamydospora P6132 P10669
Phytophthora sp canalensis P10456
Phytophthora lacustris P10337
Phytophthora humicola P3826
Phytophthora inundata P8478
Phytophthora personii P11555
Phytophthora sp sulawesiensis P6306
Phytophthora asparagi P10690
Phytophthora pseudocryptogea P3103
Phytophthora sp kelmania P10613
Phytophthora aff erythroseptica P10811
Phytophthora erythroseptica P1699
Phytophthora cryptogea P1088
Phytophthora drechsleri P10331
Phytophthora trifolii P7010
Phytophthora medicaginis P10683
Phytophthora sansomeana P3163
Phytophthora brassicae P10414
Phytophthora primulae P10333
Phytophthora sp aff brassicae P10728
Phytophthora sp aff chicorii P6207
Phytophthora austrocedri P15132
Phytophthora syringae P10330
Phytophthora hibernalis P3822
Phytophthora foliorum P10969
Phytophthora lateralis P3888
Phytophthora ramorum P10301
Phytophthora parsiana P15164
Phytophthora sp thermophilum P10457
Phytophthora hydropathica P16857
Phytophthora sp lagoariana P8223
Phytophthora sp cuyabensis P8213
Phytophthora irrigata P16861
Phytophthora sp napoensis P8221
Phytophthora macrochlamydospora P10267
Phytophthora richardiae P6875
Phytophthora quininea P3247
Phytophthora captiosa P10719
Phytophthora fallax P10725
Phytophthora insolita P6195
Phytophthora polonica P15005
Phytophthora gallica P16826
Phytophthora kernoviae P10681
Phytophthora boehmeriae P6950
Phytopythium vexans P3980
Elongisporangium undulatum P10342
0.0
0.02
